# On the Solubility and Stability of Polyvinylidene Fluoride

**DOI:** 10.3390/polym13091354

**Published:** 2021-04-21

**Authors:** Jean E. Marshall, Anna Zhenova, Samuel Roberts, Tabitha Petchey, Pengcheng Zhu, Claire E. J. Dancer, Con R. McElroy, Emma Kendrick, Vannessa Goodship

**Affiliations:** 1 WMG, International Manufacturing Centre, University of Warwick, Coventry CV4 7AL, UK; S.Roberts.7@warwick.ac.uk (S.R.); Pengcheng.zhu@warwick.ac.uk (P.Z.); C.Dancer@warwick.ac.uk (C.E.J.D.); V.Goodship@warwick.ac.uk (V.G.); 2 Department of Chemistry, University of York, York YO10 5DD, UK; anna@greenrosechemistry.com (A.Z.); tabitha.petchey@york.ac.uk (T.P.); rob.mcelroy@york.ac.uk (C.R.M.); 3 College of Engineering and Physical Sciences, University of Birmingham, Edgbaston, Birmingham B15 2TT, UK; E.Kendrick@bham.ac.uk

**Keywords:** polyvinylidene fluoride, green chemistry, polymer processing, circular economy

## Abstract

This literature review covers the solubility and processability of fluoropolymer polyvinylidine fluoride (PVDF). Fluoropolymers consist of a carbon backbone chain with multiple connected C–F bonds; they are typically nonreactive and nontoxic and have good thermal stability. Their processing, recycling and reuse are rapidly becoming more important to the circular economy as fluoropolymers find widespread application in diverse sectors including construction, automotive engineering and electronics. The partially fluorinated polymer PVDF is in strong demand in all of these areas; in addition to its desirable inertness, which is typical of most fluoropolymers, it also has a high dielectric constant and can be ferroelectric in some of its crystal phases. However, processing and reusing PVDF is a challenging task, and this is partly due to its limited solubility. This review begins with a discussion on the useful properties and applications of PVDF, followed by a discussion on the known solvents and diluents of PVDF and how it can be formed into membranes. Finally, we explore the limitations of PVDF’s chemical and thermal stability, with a discussion on conditions under which it can degrade. Our aim is to provide a condensed overview that will be of use to both chemists and engineers who need to work with PVDF.

## 1. Introduction

Fluoropolymer polyvinylidene difluoride (PVDF) is valued for its chemical and thermal inertness and is therefore in high demand across a diverse range of sectors; for example, its piezoelectric response makes it an interesting candidate in sensing applications [1,2], while its electrochemical stability and mechanical robustness means that it is of use as a binder or separator in lithium ion batteries [3,4]. The inertness of PVDF can, however, make the polymer difficult to process, because it is resistant to being dissolved in many standard organic solvents. In this review, we examine currently known solvents for PVDF, with consideration for environmental concerns in industrial PVDF processing; we also review conditions under which PVDF is unstable and will undergo chemical reactions. To our knowledge, this review is the first to unite the available data in this area, and we hope that it will therefore be of significant use for chemists and engineers working in this field.

In 2017, the global market for fluoropolymer films was estimated to be USD 1.97 billion, and a recent report estimated that this will rise to USD 2.62 billion by 2022 [5]. Fluoropolymers are highly sought after for their excellent mechanical properties, chemical inertness and good thermal resistance; in addition, some fluoropolymers demonstrate useful characteristics such as a strong piezoelectric response. The greatest share of the fluoropolymer market is for polytetrafluoroethylene (PTFE), which is most well known for its hydrophobicity and low coefficient of friction. However, PTFE is difficult to process due to its lack of solubility in all common organic solvents; other fluoropolymers with a lower degree of fluorination can show enhanced solubility while retaining sufficient chemical inertness to be useful in similar applications.

### PVDF as a Fluoropolymer

**Figure 1:**
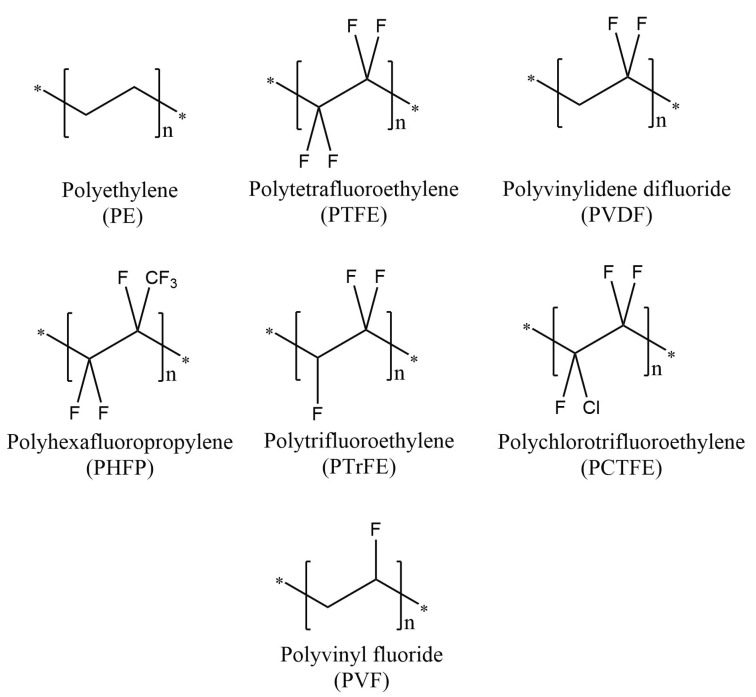
Molecular structures of fluorine-containing homopolymers. The molecular structure of polyethylene (PE), a simple hydrocarbon, is included for comparison.

The class of materials known as fluoropolymers comprises compounds in which the molecules incorporate repeating units that contain both carbon and fluorine; examples of homopolymers in this class are depicted in Figure 1 (with the structure of polyethylene also included for comparison). At a Van der Waals radius of 1.47 Å [6], the fluorine atom is small compared to other halogens but slightly larger than the hydrogen atom (radius 1.2 Å); therefore, replacing some or all of the hydrogen atoms in polyethylene (PE) with fluorine results in a stiffer polymer chain with greater resistance to bond rotation within the chain. In addition, the C–F bond is the strongest possible single bond to carbon, owing to the electronegativity of the fluorine atom (which polarises the bond, giving it significant ionic character and localising negative charge on the fluorine atom) [7]. Substituting C–F for C–H bonds within a polymer, therefore, changes the properties of the polymer considerably. Highly fluorinated polymers are known for their excellent thermal stability, UV resistance and chemical inertness along with low dielectric constant, low surface energy, low moisture absorption and low flammability [8,9].

PVDF has a similar structure to PTFE, except that the hydrogen atoms are only replaced by fluorine on every alternate carbon. This has implications for the physical properties of these polymers; both are highly unreactive compared to polyethylene due to the strength of the C–F bond, with PTFE being more unreactive than PVDF. PTFE undergoes chemical attack only under extremely harsh conditions, such as with alkali metals at high temperatures [10]; however, PVDF can undergo a few reactions using common lab reagents under somewhat milder conditions, as we shall outline in Section 3.

The degree of fluorination of the polymer also has a dramatic effect on the packing of the polymer chains in the solid state. As shown in Table 1, this results in reduced density and melting temperature of PVDF compared to PTFE. Because of this chain packing, PVDF also shows greater wettability [11] and higher coefficients of friction [12] compared to PTFE, though its wettability is still low compared to most non-fluorinated polymers. The high melting point of PTFE (>300 ${}^{{}^{\circ}}$C, [13]) implies strong cohesive forces between polymer chains. At first, this seems at odds with the anti-adhesive nature of PTFE, the property of which is usually explained in terms of very weak Van der Waals forces along PTFE chains, caused by the low polarisability of the C–F bond [14]. However, the discrepancy is explained by packing effects; the stiff, regular nature of PTFE causes it to form loose helices, which pack densely [15,16,17]; although localised Van der Waals’ forces are weak, the cumulative sum of these small interactions create a large cohesive force. At the surface of the material, the packing effect is less important compared to the weak dispersion forces and the inability of the C–F bond to participate in hydrogen bonding; thus, few materials will adhere to a PTFE surface, and water will not interact with it significantly. In PVDF, however, since only half of the carbon atoms are fluorinated, the chain has greater flexibility, packs less densely and is slightly more wettable. This effect explains both the lower melting point and lower bulk density of PVDF compared to PTFE. Interestingly, PVDF also shows a measurable glass transition temperature, while the Tg of PTFE is much less clear and a wide range of values have been quoted in the literature; one specialised study suggests that the “true” Tg is a low value [18]. Furthermore, the partial fluorination of the polymer chains in PVDF leads to a higher tensile strength than is the case for PTFE (30–70 MPa for PVDF compared to 20–30 MPa for PTFE [19]), owing to the greater proximity of C–F dipoles. The relatively high dielectric constant of PVDF (∼12 at 1 kHz [20]) also makes it an attractive candidate for some electrical applications, e.g., as a binder for electrode materials in lithium ion batteries.

The variation in polymer structure is also reflected in the solubilities of PTFE and PVDF; while PTFE is insoluble in all known organic solvents, PVDF can dissolve in some polar compounds (see Section 2 for a further discussion on solubility). PVDF can, therefore, be cast from solution or formed into membranes, while this processing method is unavailable for PTFE. In addition, PVDF behaves as a thermoplastic material and, when warmed, can thus be processed using well-established industrial techniques such as injection moulding and extrusion. PTFE, on the other hand, exhibits an extreme viscosity above its melting temperature, of the order of 10${}^{11}$ poise [23,24]; these techniques are therefore not available, and to make parts from PTFE, we must resort to either cold-machining or to sintering powdered PTFE in an appropriate mould [25,26].

**Figure 2:**
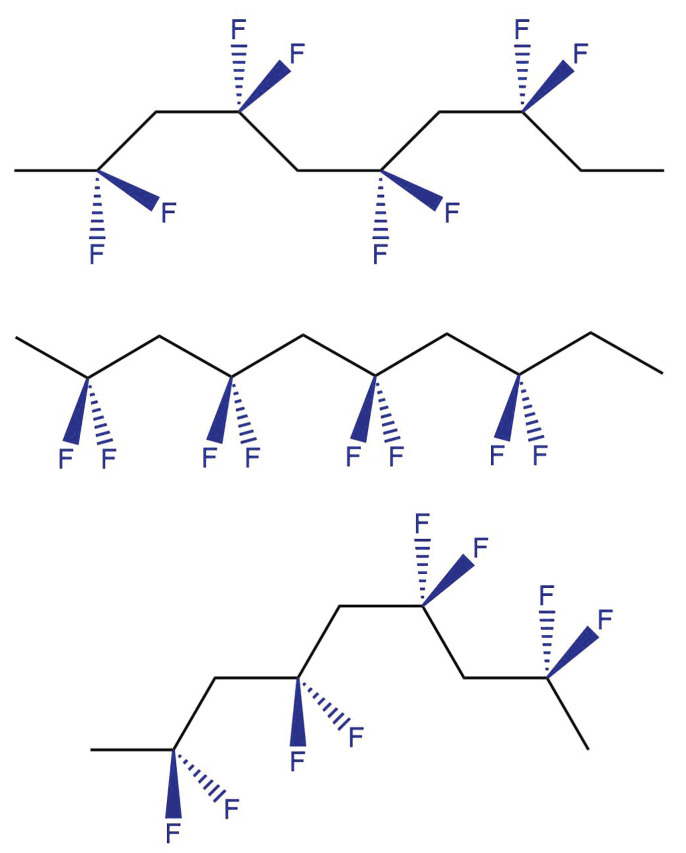
Graphic to show the molecular conformation of α (**top**), β (**centre**) and γ (**bottom**) forms of fluoropolymer polyvinylidine fluoride (PVDF). The α and γ forms contain both trans and gauche linkages, while the β form consists entirely of trans linkages along the chain backbone.

**Figure 3:**
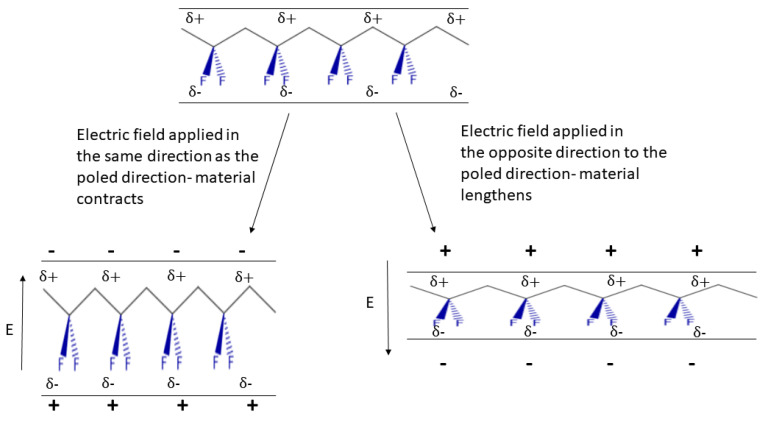
Scheme to demonstrate the piezoelectric behaviour of beta phase PVDF. The C–F bonds are aligned, with the greater electron density located on the electronegative fluorine atom. In the case where the electric field is applied in the same direction as the electrical poling, the δ− charge on the fluorine is attracted towards the positively charged electrode (and the δ+ charge on the carbon atoms is attracted towards the negatively charged electrode) and the material becomes “thicker”, therefore contracting along the direction normal to the applied field. The opposite is true when the electric field is applied in the opposite direction to the poled direction.

In addition to its effect on inter-chain bonding, the partial fluorination of the polymer backbone in PVDF means that it can crystallise into a series of different polymorphs, designated α,β,γ and δ [19,27]. PVDF is typically 50–60% crystalline, and properties such as density will depend on the extent of crystallinity and the proportion of each crystalline phase formed. The structures of the α,β and γ forms are shown in Figure 2; the δ form is rarely encountered when the material has been subjected to electric pulses at elevated temperatures [28,29]. Due to the polarisation of the C–F bond, a PVDF macromolecule can be understood to contain a large number of dipoles. The α phase is the most thermodynamically stable of the crystalline phases of PVDF but does not contain an overall preferred direction of the dipoles and, therefore, exhibits no additional response to a field. In the β and γ forms, however, it is possible for all of these small dipoles to become oriented so as to produce an overall permanent dipole. The process of aligning the dipoles is termed “poling” and involves the application of an electric field to the material while it is held above its Curie temperature, such that the chains can orient themselves with the electric field (the Curie transition of PVDF has been described as occuring over a broad range of temperatures above 170 ${}^{{}^{\circ}}$C [30]). Once aligned in this manner, the material is cooled so that the dipoles are held in their aligned position. When an electric field is applied to this material, it will distort, as shown in Figure 3, and can contract or expand in the direction normal to the applied field. Thus, the crystalline phases of PVDF can exhibit both piezoelectric and ferroelectric behaviour [31,32,33,34,35,36,37]. PVDF and its copolymers are therefore interesting candidates for use in sensing applications, and this topic has been recently reviewed [38].

We have shown, then, that PVDF is a very desirable material in applications where high purity, high tensile strength and good dielectric properties are required, coupled with a greater ease of processing than is the case for PTFE. We will now explore the options available for processing PVDF from solution.

## 2. Solubility of PVDF

The term *solvent* is in common use across a range of disciplines, but its definition is not always agreed upon. According to the International Union of Pure and Applied Chemistry (IUPAC), a solvent is defined simply as one component of a solution [39]. In this work, a PVDF solvent will be defined as a substance capable of dissolving PVDF below its melting point (roughly 160 ${}^{{}^{\circ}}$C) without chemically altering the structure of the polymer. However, in processing PVDF, temperatures above the melting point are frequently used. In these cases, the viscosity of the PVDF melt can be lowered by adding a miscible liquid known as a *diluent*. These are distinct from solvents as they dilute a melted polymer, which is typically easier than dissolving a crystalline polymer due to the flexible configuration of the melted polymer chains. As with solvents, diluents do not chemically alter the polymer but serve only to improve processability. Both solvents and diluents are important in PVDF processing.

Table 2 and Table 3 summarise known PVDF solvents and diluents, respectively, with their Hansen solubility parameters (HSPs), physical properties and references for these values. Reported uses of these compounds for PVDF processing are also listed in each table. Some compounds that act as solvents can also be used as diluents; for example, triacetin and γ-butyrolactone have been reported in both roles. Such dual-purpose compounds have been placed in the solvent table (Table 2) for conciseness, with references to both diluent and solvent studies. Compounds in Table 3 are known only as diluents, and their PVDF solvation ability below the melting point of the polymer is unreported.

While blended solvent or diluent systems are sometimes used in PVDF processing to achieve specific effects such as a uniform membrane pore structure, these blends are typically composed of an effective solvent and an ineffective one [40,41,42]. In some cases, the ineffective solvent can make up as much as 95% of the blended system but nonetheless requires a small amount of effective solvent for dissolution to occur [43]. It is worth noting that, in some cases, processing of PVDF is performed without dissolution, using non-solvents to form a slurry with a lower viscosity than that of dissolved PVDF [44]. However, in applications such as membrane casting or gel formation, solvents or diluents play critical roles in determining the morphology and behaviour of the finished product [45,46,47].

All reported solvents and diluents of PVDF fall under the *dipolar aprotic* category. These compounds have a significant permanent dipole moment and do not readily act as proton donors, limiting their reactivity [48]. While they are popular solvents for polymer dissolution as well as chemical reactions, dipolar aprotic solvents tend to present significant hazards to human health and the environment [49]. Many of the most widely used solvents in this class have therefore been restricted under the Registration, Evaluation, Authorisation and Restriction of Chemicals (REACH) legislation, creating difficulties for companies that wish to use them [50].

**Figure 4:**
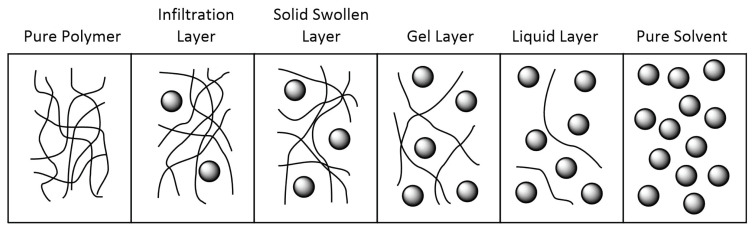
Stages of polymer dissolution, showing intermediate layers between a pure polymer and a pure solvent.

Dissolution of a polymer is governed by both enthalpy and entropy of mixing as well as kinetic effects, much like any solute. However, kinetic considerations are particularly important in polymer dissolution. Entanglement of the polymer chains hampers diffusion of the solvent into the solid polymer, resulting in a dissolution process that incorporates stages intermediate between solid and liquid (Figure 4) [227]. Initially, the solid polymer is swollen by the solvent, expanding its volume but not noticeably dissolving it. Gradually, a layer of semi-dissolved gel is formed at the solvent–polymer interface and progresses to a layer of fully dissolved liquid polymer.

As the solvent penetrates further into the polymer bulk, the proportions of these layers change; eventually the whole polymer material is dissolved. Heating the polymer mixture can greatly accelerate swelling of the polymer and diffusion of the solvent into the bulk; therefore, temperature and duration are both of great significance in PVDF dissolution. Indeed, some studies of PVDF dissolution have reported no success even with known solvents due to the study being performed with insufficient heating for too short a time [228,229].

**Figure 5:**
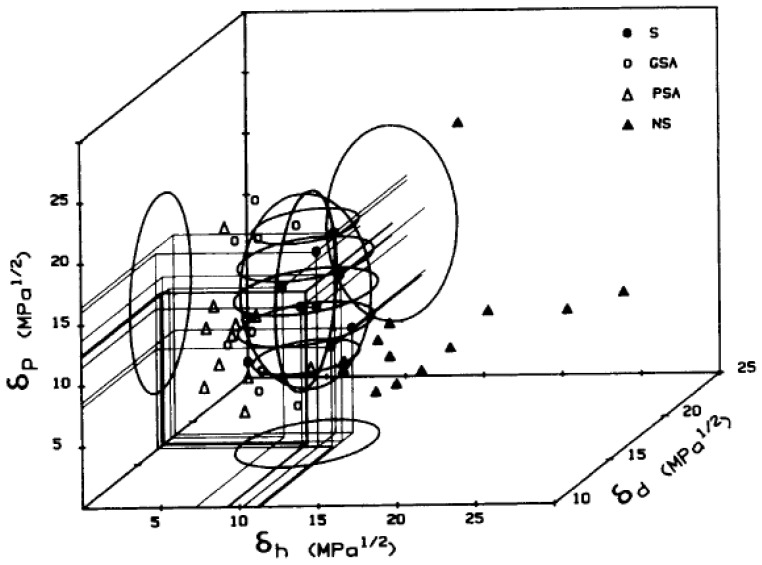
The solubility region for PVDF in Hansen space, centered on δ_D_ = 17.2, δ_P_ = 12.5, δ_H_ = 9.2. Filled circles indicate solvents, open circles indicate good swelling agents, open triangles indicate poor swelling agents, and filled triangles indicate non-solvents. (Figure reproduced with permission from [45]).

Bottino et al. conducted a comprehensive investigation of PVDF solubility in 46 solvents, generating a solubility model for this grade of PVDF ($M_{w}=4.5\times 10^{5}$ g/mol) [45]. While initial trials at 20 ${}^{{}^{\circ}}$C did not identify any successful solvents, samples were then shaken continuously at 60 ${}^{{}^{\circ}}$C for one week. By this method, eight solvents were found to dissolve PVDF, all classed as dipolar aprotics: dimethylacetamide (DMAc), *N,N*-dimethylform amide (DMF), dimethylsulfoxide (DMSO), hexamethyl phosphoramide (HMPA), *N*-methyl-2-pyrrolidinone (NMP), triethyl phosphate (TEP), trimethyl phosphate (TMP), and tetramethylurea (TMU). The solubility data were used to identify the HSPs of PVDF. HSPs provide a three-dimensional measure of solvency power based on dispersion (δ_D_), polarity (δ_P_), and hydrogen bonding (δ_H_) interactions [51]. The values of these three parameters for PVDF represent the center of a region of Hansen space in which good solvents are located; solvents falling outside this region are not likely to dissolve PVDF (Figure 5).

**Figure 6:**
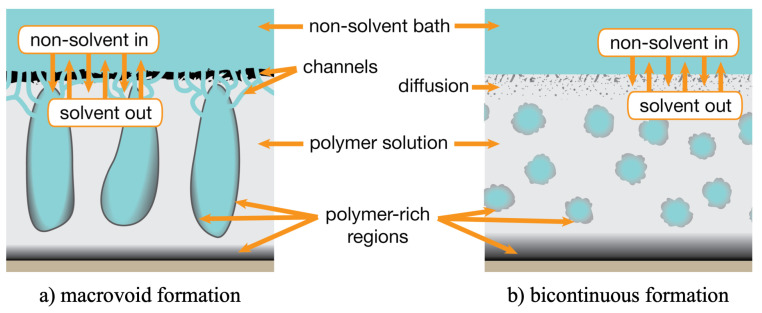
Two routes to polymer membrane formation via non-solvent induced phase separation (NIPS). (**a**) Rapid liquid–liquid demixing forms macrovoids; (**b**) Delayed demixing results in bicontinuous structure.

PVDF and other fluoropolymers are commonly used for membrane manufacture, with phase inversion methods being the most popular due to their low cost, versatility and easy scalability [230]. These methods rely on a concentrated polymer solution that undergoes demixing, forming polymer-rich and polymer-poor regions that gradually solidify into a porous membrane structure. This phase inversion can be induced in several ways, with the most widely used methods being non-solvent induced phase separation (NIPS) and thermally induced phase separation (TIPS). In NIPS, the polymer solution is immersed in a non-solvent, which gradually mixes with the solvent and forces phase separation (Figure 6). In TIPS, a solvent or diluent that can only dissolve the polymer at high temperatures is chosen, and a heated polymer solution is gradually cooled to induce phase separation [231].

In both NIPS and TIPS, the choice of solvent or diluent affects the manufacturing process and the characteristics of the resulting membrane, with small changes in solvent structure sometimes having a strong influence [230]. This has motivated a great deal of research into solvent effects on the formation of PVDF membranes, which has been recently reviewed in broader contexts [230,232,233,234].

### 2.1. Gelation of PVDF

In solvents containing a ketone moiety, PVDF is known to form thermoreversible gels, with gelation temperature and polymer chain conformation varying by solvent. Tazaki et al. studied the gelation of PVDF ($M_{w}=4.7\times 10^{5}$ g/mol) in seven ketone solvents—four linear aliphatic ketones with chain lengths from 5 to 8 as well as cycloheptanone, cyclohexanone and γ-butyrolactone (GBL) [70]. Three PVDF solvents without ketone moieties (DMAc, DMF and DMSO) were also tested and were not found to form gels.

**Figure 7:**
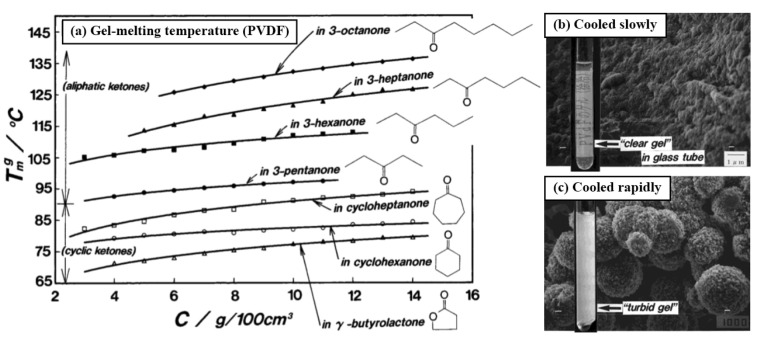
(**a**) Dependence of gel melting temperature ($T_{m}^{g}$) on polymer concentration (C) in a variety of ketone solvents; comparison of PVDF gels formed in 3-pentanone by slow cooling (**b**) vs. rapid cooling (**c**), with SEM images showing morphologies of lyophilised samples. Figure reproduced with permission from [70].

Initially, the gelling mixtures were heated to 180 ${}^{{}^{\circ}}$C, which is above the melting point of PVDF, but subsequently, the sol–gel transition was studied by heating from 30 ${}^{{}^{\circ}}$C to temperatures below 180 ${}^{{}^{\circ}}$C, showing that the compounds studied here act as solvents as well as diluents. Gelation upon cooling was found to take place rapidly in aliphatic ketones (e.g., 3-heptanone) but slowly in cyclic ketones (e.g., cycloheptanone). Gels made with cyclic ketones also tended to have lower gel melting temperatures overall ($T_{m}^{g}$), while all solvents showed an increase in $T_{m}^{g}$ with increasing polymer concentration (Figure 7a). Below 1% *w/v* polymer, precipitation occurred instead of gelation. FTIR and XRD analyses showed that PVDF samples gelled from GBL assumed γ-phase crystalline chain conformation while other ketone solvents instead yielded the α phase.

Okabe et al. explored the solution behaviour of PVDF ($M_{w}=3.1\times 10^{5}$ g/mol) in a range of solvents, including PVDF non-solvents such as hexane, ketone solvents such as 3-octanone, and strong PVDF solvents such as NMP [64]. The Flory–Huggins interaction parameter ($\chi_{12}$), which represents the magnitude of interaction between a polymer and a solvent, was estimated for PVDF in each solvent based on inverse gas chromatography (IGC) measurements. These values were then compared with the dissolution and gelation behaviour in each solvent. It was found that the two non-solvents tested, hexane and *m*-xylene, had $\chi_{12}\gg 0.5$, indicating poor solvation ability. In these solvents, PVDF precipitated into a solid when cooled from above its melting point. The strong solvents, NMP and DMAc, had $\chi_{12}\ll 0.5$, indicating good solvation ability, and maintained a single liquid phase with PVDF when cooled. For ketone and lactone solvents (3-octanone, 3-heptanone, 3-hexanone, 3-pentanone, cyclohexanone and GBL), $\chi_{12}$ was found to be close to 0.5, and these solvents formed thermoreversible gels upon cooling. In order to identify the phase of the crystallites comprising the gel’s junction points, FTIR measurements were performed with each gel. GBL, which had the lowest $\chi_{12}$ (i.e., strongest solvent–polymer interaction) of the gelling solvents, showed indications of γ-phase crystallinity when gelled. The other ketone solvents had higher $\chi_{12}$ (i.e., weaker solvent–polymer interaction) and showed α-phase crystallinity. Finally, different grades of PVDF were tested in gelling solvents, showing that grades with higher $M_{w}$ required less polymer to form a gel and that grades with higher crystallinity formed gels with higher gel–sol transition temperatures.

### 2.2. Green Solvents and Diluents for PVDF

**Figure 8:**
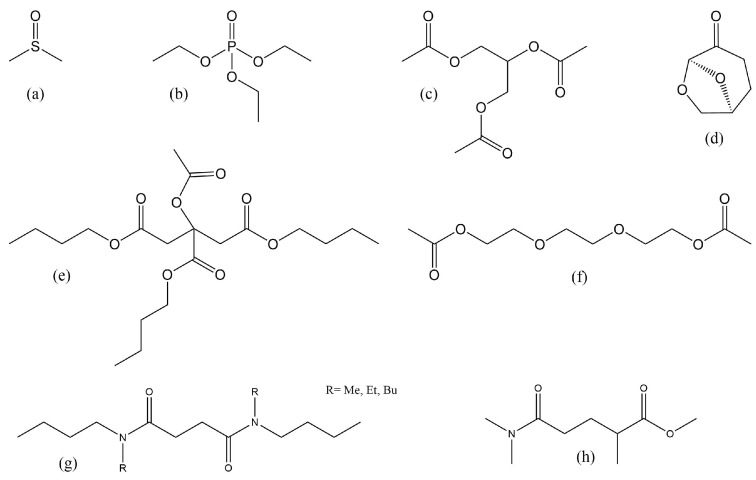
Structures of environmentally friendly PVDF solvents and diluents, including (**a**) dimethylsulfoxide (DMSO), (**b**) triethyl phosphate (TEP), (**c**) triacetin, (**d**) dihydrolevoglucosenone (Cyrene), (**e**) acetyl tributyl citrate (ATBC), (**f**) triethylene glycol diacetate (TEGDA), (**g**) *N*,*N*${}^{\prime }$-dialkyldibutylsuccindiamides and (**h**) methyl 4-(dimethylcarbamoyl)-2-methylbutanoate (Rhodiasolv^®^ Polarclean).

There has been some progress towards development of sustainable solvents and diluents for PVDF (Figure 8). Some traditional, well-studied solvents for PVDF have green characteristics, while lesser-known green compounds are emerging as sustainable alternatives. Here, we have focused on the more recent green solvents and not, e.g., ethylene carbonate. While the definition of a green solvent is complicated, the most important sustainability metrics for PVDF processing are low-hazard and bio-based (i.e., renewably sourced). All solvents readily bio-degrade unless highly halogenated, with the exception of 1,4-dioxane, which is classed as a persistent bio-accumulative toxic (PBT) due to its stability in water [126]. A comprehensive overview of solvent selection guides and definition of green solvents can be found elsewhere [235,236].

Dimethyl sulfoxide (DMSO) (Figure 8a) is a common solvent that is bio-based, has no known hazards and is registered for use at up to 100,000 tonnes annually in the EU [237]. However, it is readily absorbed through the skin and can carry toxic contaminants with it, which can be problematic in large-scale manufacturing use. Additionally, it is highly reactive and can be explosive when combined with certain halides, sodium hydride and some other substances [238]. Finally, green disposal of DMSO can be challenging, as the sulfur moiety generates SO_x_ upon incineration, and contact with water causes significant odour issues [239]. Despite these challenges, DMSO could be a suitable green solvent for specific PVDF applications, such as the preparation of microfiltration membranes. Bottino et al. tested casting PVDF membranes in eight different solvents including DMSO [46]. DMSO was identified as a suitable candidate, creating long, broad cavities in the membrane, as observed by SEM. The polymer solution was cast onto glass at 350 µm, partially evaporated in air and transferred to an non-solvent bath (cold water). In this study, PVDF membranes cast with a DMSO solvent exhibited the second highest water flux and wet membrane thickness next to hexamethylphosphoramide (HMPA). Previous studies linked thinner, finger-like pores to rapid precipitation of the polymer and hence disparity of solvent and polymer solubility parameters [240]. This suggested that diffusion was the controlling mechanism by which PVDF membranes are formed, with no significant correlation between Hansen solubility parameters and membrane structure [46]. Wang et al. concurred that the structure of the membrane sublayer was primarily influenced by the diffusion rate of the solvent and non-solvent; however, they also concluded that the top layer was in fact affected by the difference between solvent and polymer HSP [103]. On testing DMSO, DMF, DMAC, TEP and combinations of these solvents, membranes cast using DMSO or mixtures containing DMSO displayed the highest permeability owing to the relatively low diffusion rate between DMSO and water [103].

Another well-known option is triethyl phosphate (TEP) ((Figure 8b), which is used at an industrial scale as a solvent, chemical intermediate, catalyst and plastics additive. It is registered for EU use at up to 100,000 tonnes per year, and its only known hazards are eye irritation and harm by ingestion [167]. Though its hazard profile is relatively benign, large-scale use of TEP for PVDF dissolution could adversely affect world food production as phosphorus, an essential ingredient in fertiliser, becomes depleted [238]. Phosphorus is, therefore, on the critical materials list for the EU. Wang et al. prepared PVDF membranes from a variety of solvents as mentioned with respect to DMSO [103]. In their study, they showed that the solvent composition during synthesis has a physicochemical effect on the membrane structure. TEP was found to have the strongest dissolving capacity of the four solvents tested over DMF, DMAC and DMSO; however, the resultant membrane was not as porous as that prepared in DMSO. Typically, TEP requires heating to temperatures around 80 ${}^{{}^{\circ}}$C to dissolve the PVDF at 6% by weight of PVDF in solvent. The study also considered mixed solvents and found that a combination of TEP/DMSO significantly increased the surface porosity, although this membrane was soft in texture and lost some volume porosity due to the collapse of macrovoids, which consequently reduced the permeability. In a previous publication. TEP has been explored as a potential solvent for PVDF hollow fibre membrane manufacture by a dry-jet wet-spinning process [169]. In this study, Chang et al. espoused the benefits of using TEP as a safer alternative to NMP and cited a higher porosity and spongy texture. To reduce the previously observed mechanical weakness of membranes formed in TEP, the authors altered the composition of the external coagulant and bore fluid by doping with 30% TEP in water. The result was a membrane with high water flux and NaCl rejection, suggesting that TEP is still a good candidate for PVDF membrane manufacture.

Triacetin (Figure 8c), also known as glyceryl triacetate, has also been studied for its specific effectiveness in dissolving PVDF. As a non-hazardous, non-volatile liquid, triacetin is labeled as generally recognised as safe by the US Food and Drug Administration and is approved for use in food and cosmetics [241]. It is registered for EU use up to 100,000 tonnes per year [152]. Previously reported Hansen solubility parameters suggest that triacetin could be a convenient latent solvent for PVDF and is a good solvent but only when in a multi-component system, owing to similar hydrogen-bonding ability and dispersity but differing polarities [228]. It is soluble in alcohol and ether and partially soluble in water, which allows for phase separation. There have been several reports of triacetin demonstrating use as a solvent for the formation of PVDF hollow fibre membranes for the purposes of gas–liquid membrane CO_2_ and propylene absorption and separation of CO_2_ from methane. Several phase separation processes have been reviewed in the literature using different anti-solvents such as glycerol or water [161,238,242].

Unconventional and neoteric (new) solvents are also being studied for their PVDF dissolution capabilities. Dihydrolevoglucosenone (Cyrene, Figure 8d) is a novel solvent that has been investigated as a green replacement for conventional dipolar aprotic solvents. Its use as a solvent was first discovered at the University of York in 2014, and its production is currently being scaled up by the Circa Group from 50 tonnes/year in order to make it practical on an industrial scale [92,243]. Cyrene is registered under REACH for EU import and manufacture of up to 100 tonnes/year, and its only known hazard is serious eye irritation [244,245]. With efficient synthesis from cellulosic biomass, no heteroatoms aside from oxygen and ready biodegradability, Cyrene is a promising green dipolar aprotic solvent and was studied by Marino et al. for its application in PVDF membrane fabrication [95]. PVDF ($M_{w}=3.2\times 10^{5}$ g/mol) was dissolved in Cyrene at a 13% *w/v* concentration by stirring at 70 ${}^{{}^{\circ}}$C for an unspecified amount of time. This solution was used to cast PVDF membranes with bicontinuous structures, which showed promise for water filtration applications. Notably, the PVDF/Cyrene solution had a high viscosity of 810 mPa·s, which likely influenced its phase inversion rate and morphology. For comparison, similar grades of PVDF at 10% *w/v* in NMP were shown to have viscosities of 120 mPa·s ($M_{w}=2.7\times 10^{5}$ g/mol) or 350 mPa·s ($M_{w}=4.4\times 10^{5}$ g/mol) [142].

Cui et al. investigated PVDF dissolution in acetyl tributyl citrate (ATBC, Figure 8e), a low-toxicity substance that is known as a plasticiser for polymers in food contact and medical applications [177]. This compound is registered for EU use at up to 100,000 tonnes per year and has no known hazards, making it a safer alternative to traditional toxic solvents while also not depleting essential elements or causing issues upon incineration [176]. PVDF ($M_{w}=5.7\times 10^{5}$ g/mol) was found to be insoluble in ATBC at room temperature but soluble above 120 ${}^{{}^{\circ}}$C. Due to its ketone functional groups, ATBC is an effective gelling agent for PVDF and was found to form a gel between 125 and 150 ${}^{{}^{\circ}}$C at 15 wt.% polymer, 120 and 160 ${}^{{}^{\circ}}$C at 20 wt.% polymer, and 115 and 170 ${}^{{}^{\circ}}$C at 25 wt.% polymer. Though the liquid solvation range is small and only accessible at lower polymer concentrations, ATBC was found to be an effective diluent for forming PVDF membranes at high temperatures and could be particularly attractive in replacing toxic diluents for water filtration applications.

Cui et al. also investigated the use of triethylene glycol diacetate (TEGDA, Figure 8f) as another low-toxicity diluent for PVDF membrane fabrication [226]. This compound is currently known as a plasticiser and is registered for use in the EU at up to 1000 tonnes per year while having no known hazards [246]. Similar to ATBC, TEGDA contains no heteroatoms aside from oxygen, making its use and disposal more environmentally friendly than DMSO or TEP. TEGDA was tested in dissolving 10–50% *w/v* PVDF ($M_{w}=5.7\times 10^{5}$ g/mol) at 200 ${}^{{}^{\circ}}$C and, through slow cooling, was found to phase separate between 85 and 117 ${}^{{}^{\circ}}$C, depending on concentration.

Very recently, Byrne et al. developed a new class of polar aprotic solvents constituting branched diamides from the reaction of succinic acid with n-methylbutylamine, n-ethylbutylamine or dibutylamine [172]. These highly dipolar solvents with low water solubility do not show indications of toxic or mutagenic effects in preliminary testing, which is unusual for short chain amides. All three solvents were shown to dissolve 10% *w/v* PVDF ($M_{w}=1.3\times 10^{6}$ g/mol) at 80 ${}^{{}^{\circ}}$C. Upon cooling, all three systems formed strong gels.

A second short chain amide classed as a green solvent is Rhodiasolv^®^ PolarClean [247]. This solvent is not bio-derived but claimed green due to its low carbon footprint, volatility, biodegradability and the fact that it is nontoxic or mutagenic. PVDF ($M_{w}=5.7\times 10^{5}$ g/mol) at 30% *w/v* was dissolved at 160 ${}^{{}^{\circ}}$C along with other additives for the preparation of hollow fibre membranes. The PVDF was shown to crystallise out at 53 ${}^{{}^{\circ}}$C [148].

### 2.3. Supercritical Fluids for PVDF Dissolution

**Figure 9:**
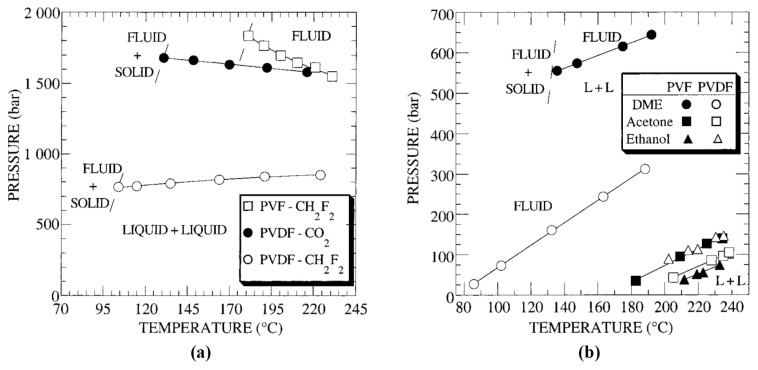
Experimental cloud-point curves for PVDF and PVF in supercritical fluids produced using (**a**) gasses at ambient conditions and (**b**) liquids at ambient conditions, with the area above each curve representing a fully dissolved single phase. The polymer concentration is 5% *w/v* in each case. Figure reproduced with permission from [224].

In addition, some studies of PVDF dissolution in supercritical fluids (SCF) have been performed. Due to the complex nature of SCF and the tunability of their properties, these substances could be classed as either solvents or diluents. They have been placed in Table 3 for the sake of simplicity. Lora et al. found that 5% *w/v* of PVDF ($M_{w}=2.0\times 10^{5}$ g/mol) could be dissolved in difluoromethane (CH_2_F_2_) at temperatures between 100 and 225 ${}^{{}^{\circ}}$C and pressures between 750 and 900 bar, while dissolution in carbon dioxide (CO_2_) required pressures above 1600 bar and temperatures of 130–215 ${}^{{}^{\circ}}$C (Figure 9a) [224]. Polar solvents dimethyl ether (DME), acetone, and ethanol were also tested, with each being capable of dissolving PVDF at relatively low pressures. These compounds were then tested as cosolvents in CO_2_, with acetone performing best. The solubility of PVDF in SCF was compared with Polyvinyl fluoride (PVF), finding that PVDF was easier to dissolve in CO_2_, CH_2_F_2_, DME and acetone but was more difficult to dissolve in ethanol (Figure 9b).

The solubility of PVDF in SCF (CO_2_ as well as halogenated solvents) was investigated further by Dinoia et al. [225]. This study concluded that, while the quadrupole moment of CO_2_ grants it sufficient polar character to act as an effective PVDF solvent, its low polarizability decreases its solvent efficacy at high temperatures. This study used a variety of PVDF grades, with $M_{w}$ ranging from $1.81\times 10^{5}$ to $3.29\times 10^{5}$, maintaining a loading of 5% *w/v* polymer across samples, and found that the molecular weight of the PVDF had only a slight effect on solubility in supercritical CO_2_. For halogenated solvents, it was found that PVDF solubility improved with increasing polarizability, dipole moment per molar volume and density of the solvent. Solubility of PVDF was then compared with a copolymer of PVDF and hexafluoropropylene. The copolymer was found to generally require lower pressures for dissolution in all SCF, which was attributed to its larger free volume.

### 2.4. Solvent Effects on PVDF Materials

Solvent choice can have an impact on properties beyond simple membrane performance. Banerjee et al. found that the resistance and capacitance values of activated carbon capacitors were highly sensitive to the solvent chosen for dissolution of the PVDF binder [104]. The solvents tested included the eight identified in Bottino’s 1988 work as well as propylene carbonate (PC). Of these, TEP produced electrodes with the highest specific capacitance and lowest internal resistance, which Banerjee attributed primarily to the low dielectric constant of this solvent. TMP, TMU, DMF and DMA also produced electrodes with high capacitance and low resistance, while the remaining solvents (HMPA, NMP, DMSO and PC) did not perform as well. Where dielectric constants between solvents were similar, improved performance was attributed to higher viscosity or lower boiling point, each of which allows for a more homogeneous distribution of particles during electrode casting and drying. Thus, careful choice of a solvent in electrode manufacturing can significantly increase the performance of the final product.

When membranes are cast using immersion in a non-solvent bath, as in NIPS, the evaporation rate of the solvent is no longer the determining factor for crystallinity. Most of the solvent is displaced by non-solvent during film formation, and therefore, evaporation rates during drying of the film are very similar [69]. In this case, polymorphism is determined by the solvent dipole moment, as the electrostatic interactions between solvent and PVDF chains alter the conformation of the chain. A study by Nishiyama et al. showed that PVDF solvents with relatively high dipole moments (PC and HMPA) favour the β phase; those with relatively low dipole moments (TEP and cyclohexanone) favour the α phase; and those with intermediate dipole moments (GBL and DMAc) exhibit a mixture of α, β and γ phases [69].

In the work by Kumar et al., HSPs were applied to identify suitable solvents for PVDF dissolution (DMSO and DMF) and for PVDF swelling (acetone, methyl ethyl ketone (MEK) and tetrahydrofuran (THF)) in order to form β and α phases, respectively [118]. Thin films were produced by dissolving/swelling PVDF in the desired solvent at 1 mg/mL loading at room temperature before casting drop wise on the water surface (air–water interface—Langmuir–Schaefer methodology) to give an oriented, single layer film. In good solvents, full dissolution of PVDF allows for rotation around the backbone of the polymer at the interface, resulting in orientation of all fluorine atoms towards water and all protons towards air. This is driven by F−OH hydrogen bonding and gives rise to a crystalline, all trans configuration: β-phase PVDF. In the swelling solvents, only the amorphous regions are solubilised, limiting the trans-configuration to these regions and leaving the polymer predominantly in the α phase.

## 3. PVDF Stability

As we have discussed, PVDF is principally in demand for its thermal and chemical stability, which are characteristic of fluoropolymers and are attributed to the strength of the C–F bond. However, there are contexts in which PVDF is used instead of the fully fluorinated PTFE, precisely because its lower degree of fluorination makes it more soluble and hence more processable. It also follows that PVDF is somewhat more reactive than PTFE; while PTFE will react chemically only under very extreme conditions, such as with elemental metals at very high temperatures [248], PVDF will react with a limited range of compounds. We discuss below the conditions under which PVDF will undergo degradation, as a useful guide to its suitable operating conditions.

### 3.1. Thermal Degradation of PVDF

**Figure 10:**
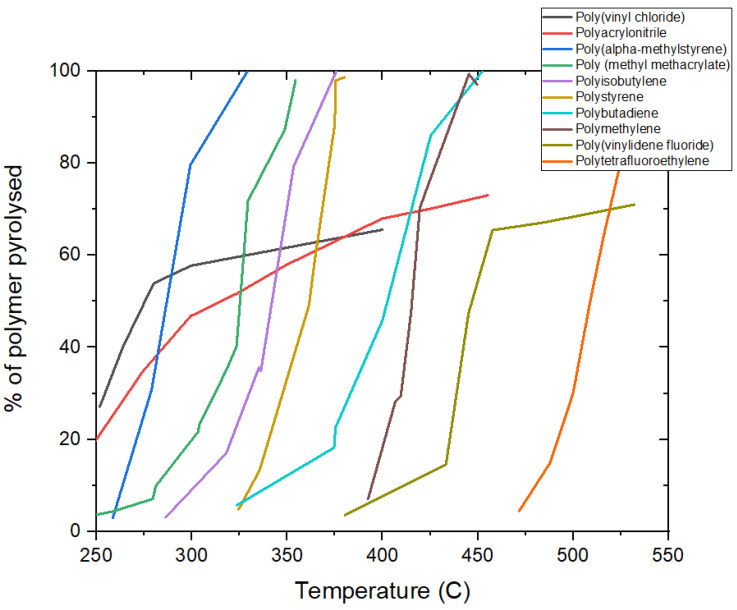
Graph to show the volatilisation of several polymers by pyrolysis under vacuum. Of this set of polymers, only polytetrafluoroethylene (PTFE) degrades at a higher temperature than PVDF. Under these conditions PVDF loses up to 60% of its mass at around 430–459 ${}^{{}^{\circ}}$C and then stabilises after this temperature. This figure is based on one from Reference [249].

By pyrolysis, it was shown in the 1950s that PVDF loses a significant fraction of its mass at around 440 ${}^{{}^{\circ}}$C, leaving a residue that is stable above this temperature [249]. Figure 10 compares this thermal behaviour with that of other common polymers; the onset of degradation for PVDF is high compared with most other polymers (except PTFE), and the mass loss stabilises after 60% of the mass is lost around 440 ${}^{{}^{\circ}}$C. These experiments were carried out under vacuum; later work showed that the exact degradation profile depends on the atmosphere to which the PVDF is exposed (air, vacuum or nitrogen) and the crystallinity of the PVDF. In 1985, Nguyen reviewed the thermal degradation pathways of PVDF and PVF [250]. It was accepted that the principal mechanism for the degradation of PVDF involves the expulsion of HF from the PVDF chain, with a concomitant formation of C=C double bonds. PVDF has actually been used as a source of F in high-temperature reactions [251,252], and therefore, any recycling process that involves pyrolysis of the PVDF must take into account the fact that HF will be produced. The stability of the residue above 440 ${}^{{}^{\circ}}$C is attributed to the C=C bonds along the backbone of the polymer chain. The hypothesis that C=C double bonds are formed is also supported by FTIR studies [253] and by the development of colouration in the chain as the double bonds form.

The presence of additives can also affect the thermal stability of PVDF. Some nanoscale filler materials have been shown to change the overall crystallinity of the material. Additives that can increase the crystallinity of the PVDF material without chemically reacting with it can enhance its thermal stability; examples of such additives include boron-doped graphene [254] and magnetite [255]. However, some additives can react with PVDF at elevated temperatures, as is the case for copper oxide and aluminium oxide nanoparticles [256,257]. Added montmorillonite filler, silica and zeolites have also been shown to reduce the thermal stability of the material [258,259]; in these cases, it is suggested that the filler reacts with the HF produced by initial PVDF degradation and that this process further advances the degradation of the PVDF.

### 3.2. Chemical Stability of PVDF

**Figure 11:**
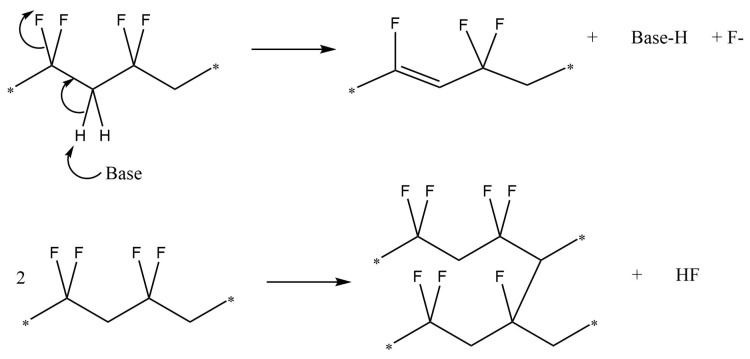
Elimination reactions undergone by PVDF, resulting in the expulsion of HF from the polymer.

Highly fluorinated polymers such as PVDF are generally rather resistant to chemical degradation. Acids seem to have very little effect on PVDF. Hydrochloric acid (HCl), humic acid (HA) and sulphuric acid (H${}_{2}$SO${}_{4}$) have been tested in varying concentrations, each showing no reaction with PVDF [260,261]. This, coupled with the dearth of literature concerning reactions of PVDF with acids suggests that PVDF is stable in low-pH environments. However, PVDF has been shown to have sensitivity to certain strong bases [262,263,264,265,266]. When exposed to strong alkaline conditions (pH ≥ 11) PVDF can readily undergo a dehydrofluorination reaction. This reaction causes an expulsion of HF and the formation of C=C double bonds or crosslinking between chains, similar to the thermal degradation reaction (see Figure 11). These conjugated double bonds allow further reaction mechanisms to occur, e.g., Diels–Alder [267] or further crosslinking [268,269]. The dehydrofluorination degradations visually appear as a colour change to yellow or brown, concurrent with the C=C double bond formation, but can also present as the gelation of a PVDF solution due to the formation of crosslinks between adjacent PVDF chains (see Figure 11). The autocatalytic formation of C=C double bonds also increases the susceptibility of neighbouring groups to dehydrofluorination [270].

**Figure 12:**
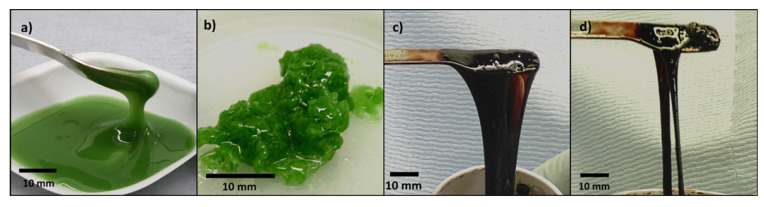
Photographs to illustrate the gelation of PVDF. (**a**) Reaction of PVDF in NMP with NiCO${}_{3}$ at 0 min (**b**) Reaction of PVDF in NMP with NiCO${}_{3}$ at 90 min. (**c**) Reaction of PVDF in NMP with NaOH at 0 min (**d**) Reaction of PVDF in NMP with NaOH at 5 min.

Given the limited available literature, several test experiments were performed in our labs to explore the stability of PVDF in mixtures intended for use in batteries. As a model system, we illustrate the dehydrofluorination of PVDF under basic conditions in a simple solution using *N*-methyl-2-pyrrolidone (NMP) as the solvent; 10 wt.% of a base (NiCO${}_{3}$ or NaOH) was added to a solution of PVDF (8 wt.% PVDF in *N*-methyl-2-pyrrolidone). The (NiCO${}_{3}$) solution (pH 8–9) gave a gel-like product after approx. 90 min, with almost no change in colour (though the mixture is already strongly green-coloured due to the presence of NiCO${}_{3}$). For the NaOH solution, however, a reaction occurred almost immediately and, within approximately 5 min, a strongly elastic gel formed. This was accompanied by a significant colour change. The PVDF solution in NMP is cloudy with a slight yellow tinge; during the reaction with NaOH, it became black/brown (see Figure 12). Subsequent tests with NaOH on an 8% PVDF/NMP solution demonstrated that, for a 10:1 molar ratio of NaOH to PVDF repeating unit, gelation occurs quickly (within 5 min) with an accompanying colour change from clear to black/brown. For a 1:1 molar ratio, the reaction occurs much more slowly. Gelation and discolouration take approximately 1 day to occur, and the solution forms a brown gel as its final product (rather than black/brown). This chemical gelation has knock-on implications for the battery industry, where highly alkaline materials such as NMC811 are emerging as key candidates for use as cathodes. The composition and preparation of PVDF inks and slurries must be carefully controlled in order to ensure their stability for coating and storage. For example, exposure to water and heat generation during the mixing and coating should be limited to stop gelation from occurring. The longer-term effect of PVDF with these alkaline materials in the cell is still not known, and the degradation or cell failure mechanisms over the lifetime may be accelerated in these alkaline cathode-containing systems. A 2018 study [271] indicates that PVDF, while stable as part of a graphite electrode, suffers from degradation if silicon is also present. Therefore, PVDF should not be treated as a completely inert material, and its stability is an important subject of study.

**Figure 13:**
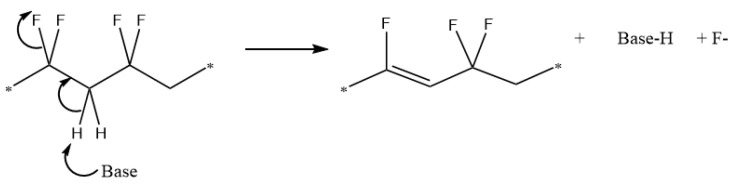
Mechanism for the elimination of HF from PVDF to form unsaturated bonds.

A dehydrofluorination reaction of PVDF dissolved in DMF upon exposure to bases was demonstrated by Dias et al. [272], with the degree of reaction dependent on the strength of the base. In similar conditions, the hydroxyl-based compounds KOH and tetramethyl-ammonium hydroxide produced gelatinous products and precipitates, respectively. While both hydroxyl-based compounds demonstrated a reaction with PVDF, the typically stronger base, KOH, gave a higher yield of precipitate (100%) compared to tetramethyl-ammonium hydroxide (10–20%). Two other bases, lithium 2,2,6,6-tetramethyl-piperidide and potassium t-butoxide, were tested and showed clear degradation in UV-VIS spectra and conductivity changes but with less visible degradation. As also demonstrated by our tests, stronger bases, such as those containing hydroxyl groups, have a higher affinity to degrading PVDF. These hydroxyl ions can react with PVDF via an elimination reaction. Nucleophilic substitution reactions are unlikely due to the relatively poor leaving-group ability of the F ion (see Figure 13 for the elimination mechanism). FTIR spectroscopy verifies the absence of OH bands in the spectra of the hydroxyl-reacted PVDF [273].

High concentrations (>30 wt.%) of NaOH have been shown to react strongly with PVDF, and this reactivity does continue with lower concentrations (<1 wt.%) as well. However, it seems that the reactivity and sensitivity of the PVDF also depend on the cation of the hydroxide. KOH does not react with PVDF in low concentrations (<1 wt.%) [274], but a reaction does occur at higher concentrations (>30 wt.%) of KOH with PVDF [266,275]. At high concentrations, KOH has a greater potential for degradation than NaOH [266]. The reactions of PVDF with hydroxides can also be catalysed by organic solvents such as tetrabutylammonium bromide (TBAB) [266,273]. TBAB is necessary for the low-concentration dehydrofluorination reaction between KOH and PVDF. However, in both cases, the reaction pathway the catalyst provides is unclear.

A discolouration reaction due to the elimination of HF in the dehydrofluorination reaction of a PVDF powder suspended in an aqueous NaOH solution was observed by Kise et al. [266]. The IR spectra demonstrated new bands at 1590 and 2100 cm${}^{-1}$, denoting C–C double and triple bonds, respectively. This dehydrofluorination reaction is consistent with the reaction where PVDF is present in solution, demonstrating that the sensitivity to bases is maintained regardless of whether the PVDF is dissolved or solid. This is important as surface area seems to has a lesser effect than strength of base on the reaction.

Super-hydrophobic PVDF membranes, made by Wu et al. [276], used hydroxyl-rich silica to induce phase inversion without degrading the PVDF. The hydroxyl groups induced a phase transition in the PVDF chains from α to γ PVDF and hydrogen bonds formed between these groups and the PVDF chains. This may suggest that, for degradation (via dehydrofluorination) to occur, the hydroxyl groups or other base anions need to be free or solvated. It is interesting to note that, as PVDF exhibits piezoelectric effects in the β and γ phases but not in the α phase [36], this phase inversion can be useful for the formation of piezoelectric materials [198,277,278].

A PVDF–OH polymer was formed during a Fenton reaction with anhydrous ethanol, hydrogen peroxide, iron (II) sulfate heptahydrate and sulphuric acid by Teow et al. [260]. In this reaction, OH radicals and OOH radicals are formed by the hydroxylation of hydrogen peroxide. These radicals then displace the hydrogens on the PVDF, forming PVDF–OH. The strong C–F bonds are unaffected, and the structure of the rest of the PVDF remains largely unchanged. It is important to note that the radicals are responsible for the reaction with PVDF, as expanded in the Irradiation Degradation section. Other reactive species have shown prevalence to reactions. Lithium silicides, due to their highly reactive nature (such as Li${}_{7}$Si${}_{3}$ and Li${}_{12}$Si${}_{7}$) can react with the lower energy bonds in PVDF, C–C and C–H [279]. This is important in the battery industry as PVDF is a commonly used binder for electrodes and silicon materials can be used as anodes; lithium silicides can form during solid electrolyte interface (SEI) formation (a layer that forms between the anode and the liquid electrolyte during the first few cycles of a lithium ion battery) and the cycling processes can further degrade the electrodes. Lithium oxides have also been shown to cause degradation of PVDF in batteries [280]. Other additives have been used in the formation of PVDF membranes and have been shown to increase the hydrophilicity of the membrane surface. These include titanium dioxide and silica.

### 3.3. Radical Reactions in PVDF

PVDF is not always inert to radiation, but high-energy radiation is required to form radicals on the PVDF chain. The radiation source can be UV [281], electron beam [282], heavy ions or gamma radiation [283]. Once irradiated, radicals are formed by bond scission between the carbon chain and the H or F substituents on the chain. These radicals can form crosslinks between neighbouring chains, create unsaturated bonds within the PVDF structure or aid grafting reactions.

For the synthesis of graft copolymers, radical formation is beneficial; however, in most situations, it is undesirable. Exposure to UV radiation can cause cracks and fractures on the surface of PVDF membranes, and the extent of damage is observed to increase with further exposure. Additionally, the exposure can cause a reduction in tensile strength; a reduction of 10% after 120 h was observed by Lee et al. [281]. The study concluded that, under short time scales (<40 h), the effect of UV radiation on PVDF was minimal but that prolonged exposure critically altered the membrane. This has clear implications for the use of PVDF in environments where UV exposure is inevitable, e.g., in aerospace applications.

Electron beam (EB) radiation has been shown to increase the crosslinking within the PVDF [284,285] and to thereby reduce the size of pores within PVDF membranes. Critically, this allows a PVDF membrane to be used as a filter for smaller particles, although the degree of pore homogeneity was greatly reduced with exposure to radiation. However, the changes to the surface roughness and surface functionalisation caused by the radiation led to an increase in hydrophilicity and a decrease in water flux [286]. Additionally, the EB radiation can reduce the tensile strength of the films due to chain scission [287]. γ-radiation has been similarly demonstrated to cause chain scission in cross-linked structures, such as by Medeiros et al. [288]. Owing to PVDF’s wastewater filtration uses, radiation changes of this nature are a potential problem.

## 4. Summary

The vast range of commercial applications that currently make use of PVDF spans industries as diverse as the chemical, medical, automotive, semiconductor and food packaging sectors (among many others). With our current growing need for energy storage, its use as a binder in battery electrode materials is of particular interest and will only become more important as the manufacture of electric vehicles increases. As the consumption of PVDF becomes more important, however, it is important to consider how it can be recycled or reused as PVDF components reach the end of their useful lives. This is particularly urgent when PVDF is used as a binder in electrode materials because, in that application, it is generally mixed with a number of other materials. The thermal stability and chemical inertness of PVDF (which are the very qualities that make it desirable in many areas) render it difficult in being reclaimed from a composite, especially as it is soluble in few solvents. A further environmental consideration here is that disposal of PVDF by incineration leads to the formation of HF at elevated temperatures. The aim of this review is to provide a useful reference for both chemists and engineers who wish to make use of PVDF and to initiate discussion on the role that PVDF may play in a future circular economy.

In Section 1, we discuss how the beneficial material properties of PVDF (such as thermal and chemical stability) make it a desirable material and have led to its use in the wide variety of applications mentioned above. To enable greater flexibility for processing and recycling of PVDF materials, greater knowledge in alternative and “green” solvents is required, as currently there are limitations for current solvents. The current state-of-the-art is summarised in Section 2. An overview of the available literature on known solvents of PVDF, with a discussion of PVDF membrane formation, is provided. A current “map” of solvents and diluents for PVDF with reference to their Hansen solubility parameters is presented. There are significant future research opportunities to further explore the applicability of “green” solvents, solvent mixtures and supercritical fluids for PVDF.

Finally, we discuss the limitations of PVDF’s stability (Section 3). In strong bases and under strong radiation, it is possible to see degradation of the polymer and it is important for the engineer to understand possible constraints on the practical use of the material. This becomes particularly important for example in the battery and fuel cell industry, where PVDF is used as a binding agent in electrodes and for the synthesis of membranes. Alkaline materials within the composites may produce instabilities in inks or coatings and provide routes for degradation during a product lifetime. The precise degradation mechanisms and their impact is still largely unexplored.

In summary, this article provides insight into how the use of PVDF may be extended by good knowledge of the solubility of the material and how it may be processed; it is also useful to understand the limitations of PVDF by exploring situations in which it may undergo degradation. There are many future opportunities to enable greater processability and functionality through new combinations of solvents. There is also considerable scope for further investigation of PVDF’s degradation mechanisms under different conditions.

## Figures and Tables

**Table 1:** Table of fluoropolymer material properties. Data on High Density Polyethylene (HDPE) are included for comparison. Data are taken from [13,21,22].

Polymer	Density (g cm${}^{-3}$)	Melting Temperature (${}^{{}^{\circ}}$C)	Glass Transition Temperature (${}^{{}^{\circ}}$C)
PTFE	2.16–2.20	317–345	
PVDF	1.76–1.83	158–200	−29 to −57
PCTFE	2.1–2.2	210	45
PVF	1.34	190	−15 to −20 and 40 to 50
HDPE	0.94–0.965	125–135	−118 to −133

**Table 2:** Known solvents for PVDF. All measurements are at 25 ${}^{{}^{\circ}}$C and 760 mmHg unless noted otherwise. δ_D_, δ_P_, δ_H_ = Hansen solubility parameters, MP = melting point, BP = boiling point, FP = flash point, ρ = density, η = dynamic viscosity, and γ = surface tension.

	δ_D_	δ_P_	δ_H_	MP	BP	FP	ρ	η	γ	References	
**Solvent**	**MPa^0.5^**			**${}^{{}^{\circ}}$C**			**g/cm^3^**	**mPa**·s	**mN/m**	**Properties**	**Use**
Acetone ${}^{a}$	15.5	10.4	7.0	−95	56	−17	0.79	0.3	23.5	[51,52,53]	[54,55,56,57]
Acetyl triethyl citrate (ATEC) ${}^{b}$	16.6	3.5	8.6	−45	228 ${}^{c}$	188	1.14	53.7		[51,58]	[59]
γ−Butyrolactone (GBL) ${}^{a,b}$	19.0	16.6	7.4	−45	204	98	1.12	2.0	44.6	[51,60,61]	[62,63,64,65,66,67,68,69,70]
Cyclohexanone (CHO)	17.8	6.3	5.1	−31	154	44	0.95	2.2	35.1	[51,71,72]	[64,69,70,73]
Cyclopentanone (CPO) ${}^{a}$	17.9	11.9	5.2	−51	131	30	0.94	1.1	33.8	[51,74]	[75]
Dibutyl phthalate (DBP) ${}^{b}$	17.8	8.6	4.1	−35	339	171	1.05	19.7	33.4	[51,76,77]	[40,41,66,78,79,80,81,82,83]
Dibutyl sebacate (DBS)	13.9	4.5	4.1	−10	345	178	0.94	8.0	33.1	[51,84,85]	[66]
Diethyl carbonate (DEC) ${}^{a}$	16.6	3.1	6.1	−43	126	25	0.98	0.8	26.8	[51,86,87]	[68]
Diethyl phthalate (DEP) ${}^{b}$	17.6	9.6	4.5	−60	297	170	1.12	12.9	23.5	[51,88,89]	[90,91]
Dihydrolevoglucosenone (Cyrene) ${}^{a}$	18.8	10.6	6.9	−20	227	108	1.25	14.5	72.5	[92,93,94]	[95]
Dimethylacetamide (DMAc)	16.8	11.5	10.2	−20	166	64	0.94	0.9	32.4	[51,76,96]	[45,46,54,56,64,69,73,97,98,99,100,101,102,103,104,105,106,107]
*N,N*−dimethylformamide (DMF)	17.4	16.7	11.3	−61	153	58	0.94	0.9	35.2	[51,108,109]	[45,46,55,57,65,98,99,102,103,104,107,110,111,112,113,114,115,116,117,118,119]
Dimethylsulfoxide (DMSO) ${}^{a}$	18.4	16.4	10.2	19	189	87	1.10	1.9	42.7	[51,120,121]	[45,46,55,99,103,104,107,118,122,123,124]
1,4−Dioxane	19.0	1.8	7.4	12	101	11	1.03	1.3	32.7	[51,125,126]	[127]
3−Heptanone ${}^{b}$	16.2	5.0	4.1	−39	146	41	0.81	0.8	25.7	[51,128,129,130]	[64,70]
Hexamethyl phosphoramide (HMPA)	18.5	8.6	11.3	7	231 ${}^{d}$	144	1.03	3.5	34.4	[51,131,132]	[45,46,54,56,69,104,114,116]
3−Hexanone	15.7	6.7	4.1		−56	124	18	0.82		[51,133,134,135]	[64,70]
Methyl ethyl ketone (MEK)	16.0	9.0	5.1	−86	80	−9	0.81	0.4	24.3	[51,136,137]	[55,57]
*N*−methyl−2−pyrrolidinone (NMP)	18.0	12.3	7.2	−24	204	91	1.03	1.7	40.3	[51,138,139]	[45,46,57,64,98,102,104,107,114,117,119,124,140,141,142,143,144]
3−Octanone ${}^{b}$	16.2	4.5	4.1	−23	169	53	0.82		26.2	[51,129,145]	[64,70]
Rhodiasolv^®^ PolarClean${}^{a}$	17.2	8.6	9.7	−60	278	144	1.04	7.4	37.5	[146,147]	[148,149]
3−Pentanone ${}^{b}$	15.8	7.6	4.7	−39	100	13	0.81	0.4	24.7	[51,150,151]	[64,70]
Propylene carbonate (PC) ${}^{a}$	20.0	18.0	4.1	−49	242	116	1.20	2.8	31.9	[51,152,153]	[66,68,69,81,104]
Tetrahydrofuran (THF)	16.8	5.7	8.0	−108	65	−21	0.88	0.5	27.1	[51,154,155]	[55]
Tetramethylurea (TMU)	16.7	8.2	11.0	−1	177	75	0.97	1.4	34.6	[51,156,157,158]	[45,46,104]
Triacetin ${}^{a,b}$	16.5	4.5	9.1	−78	258	148	1.16	22.5	35.2	[51,159,160]	[90,161,162,163,164]
Triethyl citrate (TEC)	16.5	4.9	12.0	−40	287	178	1.14	32.2	41.5	[51,165]	[59]
Triethyl phosphate (TEP) ${}^{a}$	16.7	11.4	9.2	−56	216	115	1.07	1.8	29.6	[51,129,166,167]	[45,46,69,98,103,104,116,168,169,170]
Trimethyl phosphate (TMP)	16.7	15.9	10.2	−46	197	107	1.20	2.3	37.0	[51,129,166,171]	[45,46,101,104,116]
*N*,*N*${}^{\prime }$ tetrabutylsuccindiamide (TBSA) ${}^{a}$	17.2	9.0	2.9	−76	>250		0.96			[172]	[172]

${}^{a}$ Green solvent, ${}^{b}$ also used as a diluent, ${}^{c}$ measured at 100 mm Hg, and ${}^{d}$ measured at 740 mmHg.

**Table 3:** Known diluents for PVDF. All measurements are at 25 ${}^{{}^{\circ}}$C and 760 mmHg unless noted otherwise. δ_D_, δ_P_, δ_H_ = Hansen solubility parameters, MP = melting point, BP = boiling point, FP = flash point, ρ = density, η = dynamic viscosity, and γ = surface tension. [BMIm][BF4] = 1-Butyl-3-methylimidazolium tetrafluoroborate.

	δ_D_	δ_P_	δ_H_	MP	BP	FP	ρ	η	γ	References	
**Solvent**	**MPa^0.5^**			**${}^{{}^{\circ}}$C**			**g/cm^3^**	**mPa**·s	**mN/m**	**Properties**	**Use**
Acetophenone	19.6	8.6	3.7	20	202	105	1.03	1.7	39.0	[51,129,173]	[47,174,175]
Acetyl tributyl citrate (ATBC) ${}^{a}$	16.7	2.5	7.4	−80	331 ${}^{b}$	218	1.05	42.5	54.6	[51,176]	[59,177,178]
Benzophenone	19.6	8.6	5.7	48	305	138	1.11		40.7	[51,129,179]	[81,180,181,182,183]
[BMIm][BF4]						288	1.21	93.8	43.0	[184,185,186]	[187]
ϵ-Caprolactam (CPL)	19.4	13.8	3.9	69	271	152	1.10		49.4	[51,129,188,189]	[190]
Cycloheptanone	17.2	10.6	4.8	−21	179	56	0.95		34.1	[51,129,191,192]	[70]
Dimethyl adipate	16.3	6.8	8.5	9	115 ${}^{c}$	107	1.06	3.0	29.1	[146,193]	[194,195]
Diethyl azelate ${}^{a}$	16.1	4.4	5.1	−19	292	>113	0.97			[146,196,197]	[195,198]
Diethyl glutarate ${}^{a}$	16.3	7.0	7.8	−24	237	96	1.02			[146,199,200]	[198]
Diethyl malonate ${}^{a}$	16.1	7.7	8.3	−20	197	90	1.06	1.7	31.3	[51,129,201,202]	[198]
Diethyl oxalate ${}^{a}$	16.2	8.0	8.8	−39	186	76	1.08	2.0	32.2	[51,76,203]	[198]
Diethyl pimelate	16.2	4.7	5.8	−24	254	113	0.99			[146,204,205]	[198]
Diethyl succinate ${}^{a}$	16.2	6.8	8.7	−29	217	98	1.04	2.7	30.6	[146,206,207]	[195,198]
Dimethyl phthalate (DMP)	18.6	10.8	4.9	0	283	154	1.19	17.2	41.2	[51,129,208]	[78,81,209]
Dimethyl sulfone	19.0	19.4	12.3	105	280	139	0.82			[51,210]	[211]
Diphenyl carbonate (DPC)	17.0	3.9	3.2	83	306	168	1.12			[146,212,213]	[182]
Ethyl benzoate (EB)	17.9	6.2	6.0	−33	212	88	1.04	2.2	34.9	[51,129,214]	[47,174,175]
Glyceryl tributyrate (GTB)	16.3	2.5	7.0	−75	307	180	1.03	10.4		[146,150,215,216]	[174,175,217]
Methyl salicylate	18.1	8.0	13.9	−9	221	96	1.18	1.5	39.2	[51,129,218]	[219]
Sulfolane	20.3	18.2	10.9	28	285	176	1.26	10.3	35.5	[51,76,220]	[221]
Supercritical fluids ${}^{a}$	15.6	5.2	5.8				0.46–0.86	0.06–0.12		[51,222,223]	[224,225]
Triethylene glycol diacetate (TEGDA) ${}^{a}$	16.5	6.0	8.2	−57	295	174	1.12	10.1		[146]	[226]

${}^{a}$ Green solvent, ${}^{b}$ measured at 732 mmHg, and ${}^{c}$ measured at 13 mmHg.

## References

[1] Wei J., Wang Z., Xing X. (2020). A wireless high-sensitivity fetal heart sound monitoring system. Sensors.

[2] Maity K., Garain S., Henkel K., Schmeisser D., Mandal D. (2020). Self-Powered Human-Health Monitoring through Aligned PVDF Nanofibers Interfaced Skin-Interactive Piezoelectric Sensor. ACS Appl. Polym. Mater..

[3] Yi S., Xu T., Li L., Gao M., Du K., Zhao H., Bai Y. (2020). Fast ion conductor modified double-polymer (PVDF and PEO) matrix electrolyte for solid lithium-ion batteries. Solid State Ionics.

[4] Barbosa J.C., Dias J.P., Lanceros-Méndez S., Costa C.M. (2018). Recent advances in poly(Vinylidene fluoride) and its copolymers for lithium-ion battery separators. Membranes.

[5] Research M. (2019). Worldwide Fluoropolymer Market Analysis & Forecast (2013–2023) Opportunities & Forecast (2019 Edition)—ResearchAndMarkets.com.

[6] Batsanov S.S. (2001). Van der Waals radii of elements. Inorg. Mater..

[7] O’Hagan D. (2008). Understanding organofluorine chemistry. An introduction to the C–F bond. Chem. Soc. Rev..

[8] Feiring A., Buschow K.J., Cahn R.W., Flemings M.C., Ilschner B., Kramer E.J., Mahajan S., Veyssière P. (2001). Fluorine-containing Polymers. Encyclopedia of Materials: Science and Technology.

[9] Peng H. (2019). Synthesis and Application of Fluorine-Containing Polymers with Low Surface Energy. Polym. Rev..

[10] Tasker S., Chambers R.D., Badyal J.P.S. (1994). Surface Defluorination of PTFE by Sodium Atoms. J. Phys. Chem..

[11] Lee S., Park J.S., Lee T.R. (2008). The Wettability of Fluoropolymer Surfaces: Influence of Surface Dipoles. Langmuir.

[12] Quaglini V., Dubini P. (2011). Friction of polymers sliding on smooth surfaces. Adv. Tribol..

[13] Wypych G. (2016). Handbook of Polymers.

[14] Stark A.Y., Dryden D.M., Olderman J., Peterson K.A., Niewiarowski P.H., French R.H., Dhinojwala A. (2015). Adhesive interactions of geckos with wet and dry fluoropolymer substrates. J. R. Soc. Interface.

[15] Sprik M., Rothlisberger U., Klein M.L. (1999). Conformational and orientational order and disorder in solid polytetrafluoroethylene. Mol. Phys..

[16] Quarti C., Milani A., Castiglioni C. (2013). Ab Initio Calculation of the IR Spectrum of PTFE: Helical Symmetry and Defects. J. Phys. Chem. B.

[17] Soper A.K., Page K., Llobet A. (2013). Empirical potential structure refinement of semi-crystalline polymer systems: Polytetrafluoroethylene and polychlorotrifluoroethylene. J. Phys. Condens. Matter.

[18] Calleja G., Jourdan A., Ameduri B., Habas J.P. (2013). Where is the glass transition temperature of poly(tetrafluoroethylene)? A new approach by dynamic rheometry and mechanical tests. Eur. Polym. J..

[19] Cardoso V.F., Correia D.M., Ribeiro C., Fernandes M.M., Lanceros-Méndez S. (2018). Fluorinated Polymers as Smart Materials for Advanced Biomedical Applications. Polymers.

[20] Chiang C.K., Popielarz R. (2002). Polymer Composites with High Dielectric Constant. Ferroelectrics.

[21] Ebnesajjad S. (2013). Introduction to Fluoropolymers: Materials, Technology, and Applications.

[22] Ibeh C.C. (2011). Thermoplastic Materials: Properties, Manufacturing Methods, and Applications.

[23] Sperati C.A., Starkweather H.W. (1961). Fluorine-containing polymers. II. Polytetrafluoroethylene. Fortschritte Der Hochpolymeren-Forschung.

[24] Hanford W.E., Joyce R.M. (1946). Polytetrafluoroethylene. J. Am. Chem. Soc..

[25] Voaden A.T. (1974). Powder Moulding Process. UK Patent.

[26] (1978). Method of Moulding Polytetrafluoroethylene. UK Patent.

[27] Nalwa H.S. (1995). Ferroelectric Polymers: Chemistry, Physics, and Applications.

[28] Li M., Wondergem H.J., Spijkman M.J., Asadi K., Katsouras I., Blom P.W.M., De Leeuw D.M. (2013). Revisiting the *δ*-phase of poly(vinylidene fluoride) for solution-processed ferroelectric thin films. Nat. Mater..

[29] Martín J., Zhao D., Lenz T., Katsouras I., De Leeuw D.M., Stingelin N. (2017). Solid-state-processing of *δ*-PVDF. Mater. Horiz..

[30] Lovinger A.J., Davis D.D., Cais R.E., Kometani J.M. (1986). On the Curie temperature of poly(vinylidene fluoride). Macromolecules.

[31] Martins P., Lopes A., Lanceros-Mendez S. (2014). Electroactive phases of poly(vinylidene fluoride): Determination, processing and applications. Prog. Polym. Sci..

[32] Kawai H. (1969). The Piezoelectricity of Poly (vinylidene Fluoride). Jpn. J. Appl. Phys..

[33] Sessler G. (1981). Piezoelectricity in Polyvinylidene Fluoride. J. Acoust. Soc. Am..

[34] Abdalla S., Obaid A., Al-Marzouki F. (2016). Preparation and characterization of poly(vinylidene fluoride): A high dielectric performance nano-composite for electrical storage. Results Phys..

[35] Broadhurst M.G., Davis G.T. (1984). Physical basis for piezoelectricity in PVDF. Ferroelectrics.

[36] Bera B., Das Sarkar M. (2017). Piezoelectricity in PVDF and PVDF Based Piezoelectric Nanogenerator: A Concept. IOSR J. Appl. Phys..

[37] Sappati K.K., Bhadra S. (2018). Piezoelectric Polymer and Paper Substrates: A Review. Sensors.

[38] Wang T., Farajollahi M., Choi Y.S., Lin I.T., Marshall J.E., Thompson N.M., Kar-Narayan S., Madden J.D.W., Smoukov S.K. (2016). Electroactive polymers for sensing. Interface Focus.

[39] McNaught A., Wilkinson A. (1997). IUPAC Compendium of Chemical Terminology.

[40] Li X., Xu G., Lu X., Xiao C. (2008). Effects of Mixed Diluent Compositions on Poly(Vinylidene Fluoride) Membrane Morphology in a Thermally Induced Phase-Separation Process. J. Appl. Polym. Sci..

[41] Zhou Q., Wang Z., Shen H., Zhu Z., Liu L., Yang L., Cheng L. (2016). Morphology and Performance of PVDF TIPS Microfiltration Hollow Fiber Membranes Prepared from PVDF/DBP/DOP Systems for Industrial Application. J. Chem. Technol. Biotechnol..

[42] Wang Z., Tang Y., Li B. (2018). Bicontinuous and Cellular Structure Design of PVDF Membranes by Using Binary Solvents for the Membrane Distillation Process. RSC Adv..

[43] Dornbusch M., Erk C., Karl U., Mizuno H. (2017). Polyvinylidene Fluoride Solutions in N-Formyl or N-Acetylmorpholine. European Patent.

[44] Kosar W.P. (2018). Pvdf Powder for Liquid Slurries. US Patent.

[45] Bottino A., Capannelli G., Munari S., Turturro A. (1988). Solubility parameters of poly(vinylidene fluoride). J. Polym. Sci. Part B Polym. Phys..

[46] Bottino A., Camera-Roda G., Capannelli G., Munari S. (1991). The Formation of Microporous Polyvinylidene Difluoride Membranes by Phase Separation. J. Membr. Sci..

[47] Mal S., Maiti P., Nandi A.K. (1995). On the Gelation Rates of Thermoreversible Poly(Vinylidene Fluoride) Gels. Macromolecules.

[48] Buncel E., Stairs R., Wilson H. (2003). The Role of the Solvent in Chemical Reactions.

[49] Diorazio L.J., Hose D.R.J., Adlington N.K. (2016). Toward a More Holistic Framework for Solvent Selection. Org. Process Res. Dev..

[50] Bergkamp L., Herbatschek N. (2014). Regulating Chemical Substances under REACH: The Choice between Authorization and Restriction and the Case of Dipolar Aprotic Solvents: Regulating Chemical Substances Under Reach. Rev. Eur. Comp. Int. Environ. Law.

[51] Hansen C.M. (2007). Hansen Solubility Parameters: A User’s Handbook.

[52] European Chemicals Agency (2020). Acetone Registration Dossier. https://echa.europa.eu/registration-dossier/-/registered-dossier/15460.

[53] Egemen E., Nirmalakhandan N., Trevizo C. (2000). Predicting Surface Tension of Liquid Organic Solvents. Environ. Sci. Technol..

[54] Kobayashi M., Tashiro K., Tadokoro H. (1975). Molecular Vibrations of Three Crystal Forms of Poly(Vinylidene Fluoride). Macromolecules.

[55] Park Y.J., Kang Y.S., Park C. (2005). Micropatterning of Semicrystalline Poly(Vinylidene Fluoride) (PVDF) Solutions. Eur. Polym. J..

[56] Horibe H., Sasaki Y., Oshiro H., Hosokawa Y., Kono A., Takahashi S., Nishiyama T. (2014). Quantification of the Solvent Evaporation Rate during the Production of Three PVDF Crystalline Structure Types by Solvent Casting. Polym. J..

[57] Zhu H., Matsui J., Yamamoto S., Miyashita T., Mitsuishi M. (2015). Solvent-Dependent Properties of Poly(Vinylidene Fluoride) Monolayers at the Air–Water Interface. Soft Matter.

[58] European Chemicals Agency (2020). Triethyl O-Acetylcitrate Registration Dossier. https://echa.europa.eu/registration-dossier/-/registered-dossier/23424.

[59] Sawada S.i., Ursino C., Galiano F., Simone S., Drioli E., Figoli A. (2015). Effect of Citrate-Based Non-Toxic Solvents on Poly(Vinylidene Fluoride) Membrane Preparation via Thermally Induced Phase Separation. J. Membr. Sci..

[60] Schwarz W., Schossig J. (2005). Butyrolactone. Ullmann’s Encyclopedia of Industrial Chemistry.

[61] European Chemicals Agency (2020). γ-Butyrolactone Registration Dossier. https://echa.europa.eu/registration-dossier/-/registered-dossier/14990.

[62] Cho J.W., Song H.Y., Kim S.Y. (1993). Thermoreversible Gelation of Poly(Vinylidene Fluoride) in *γ*-Butyrolactone Solution. Polymer.

[63] Cho J.W., Lee G.W. (1996). Thermoreversible Gelation of Blend of Poly(Vinylidene Fluoride) and Poly(Vinylidene Fluoride-Trifluoroethylene) in *γ*-Butyrolactone Solution. J. Polym. Sci. Part B Polym. Phys..

[64] Okabe M., Wada R., Tazaki M., Homma T. (2003). The Flory-Huggins Interaction Parameter and Thermoreversible Gelation of Poly(Vinylidene Fluoride) in Organic Solvents. Polym. J..

[65] Ogoshi T., Chujo Y. (2005). Synthesis of Poly(Vinylidene Fluoride) (PVdF)/Silica Hybrids Having Interpenetrating Polymer Network Structure by Using Crystallization between PVdF Chains. J. Polym. Sci. Part A Polym. Chem..

[66] Su Y., Chen C., Li Y., Li J. (2007). PVDF Membrane Formation via Thermally Induced Phase Separation. J. Macromol. Sci. Part A.

[67] Cha B.J., Yang J.M. (2007). Preparation of Poly(Vinylidene Fluoride) Hollow Fiber Membranes for Microfiltration Using Modified TIPS Process. J. Membr. Sci..

[68] Shimizu H., Arioka Y., Ogawa M., Wada R., Okabe M. (2011). Sol-Gel Transitions of Poly(Vinylidene Fluoride) in Organic Solvents Containing LiBF_4_. Polym. J..

[69] Nishiyama T., Sumihara T., Sasaki Y., Sato E., Yamato M., Horibe H. (2016). Crystalline Structure Control of Poly(Vinylidene Fluoride) Films with the Antisolvent Addition Method. Polym. J..

[70] Tazaki M., Wada R., Abe M.O., Homma T. (1997). Crystallization and Gelation of Poly(Vinylidene Fluoride) in Organic Solvents. J. Appl. Polym. Sci..

[71] Korosi G., Kovats E.S. (1981). Density and Surface Tension of 83 Organic Liquids. J. Chem. Eng. Data.

[72] European Chemicals Agency (2020). Cyclohexanone Registration Dossier. https://echa.europa.eu/registration-dossier/-/registered-dossier/15388.

[73] Salimi A., Yousefi A.A. (2004). Conformational Changes and Phase Transformation Mechanisms in PVDF Solution-Cast Films. J. Polym. Sci. Part B Polym. Phys..

[74] European Chemicals Agency (2020). Cyclopentanone Registration Dossier. https://echa.europa.eu/registration-dossier/-/registered-dossier/13920.

[75] Ardel G., Golodnitsky D., Freedman K., Peled E., Appetecchi G.B., Romagnoli P., Scrosati B. (2002). Rechargeable Lithium/Hybrid-Electrolyte/Pyrite Battery. J. Power Sources.

[76] Wang J., Du H., Liu H., Yao X., Hu Z., Fan B. (2007). Prediction of Surface Tension for Common Compounds Based on Novel Methods Using Heuristic Method and Support Vector Machine. Talanta.

[77] European Chemicals Agency (2020). Dibutyl Phthalate Registration Dossier. https://echa.europa.eu/registration-dossier/-/registered-dossier/14862.

[78] Gu M., Zhang J., Wang X., Tao H., Ge L. (2006). Formation of Poly(Vinylidene Fluoride) (PVDF) Membranes via Thermally Induced Phase Separation. Desalination.

[79] Li X., Lu X. (2006). Morphology of Polyvinylidene Fluoride and Its Blend in Thermally Induced Phase Separation Process. J. Appl. Polym. Sci..

[80] Ji G.L., Du C.H., Zhu B.K., Xu Y.Y. (2007). Preparation of Porous PVDF Membrane via Thermally Induced Phase Separation with Diluent Mixture of DBP and DEHP. J. Appl. Polym. Sci..

[81] Yang J., Wang X.L., Tian Y., Lin Y., Tian F. (2010). Morphologies and Crystalline Forms of Polyvinylidene Fluoride Membranes Prepared in Different Diluents by Thermally Induced Phase Separation. J. Polym. Sci. Part B Polym. Phys..

[82] Cui A., Liu Z., Xiao C., Zhang Y. (2010). Effect of Micro-Sized SiO2-Particle on the Performance of PVDF Blend Membranes via TIPS. J. Membr. Sci..

[83] Song Z., Yang W., Zhang J., Li Y., Yuan S. (2015). Fabrication of Hollow Fiber Microfiltration Membrane from PVDF/DBP/DBS System via Thermally Induced Phase Separation Process. J. Polym. Eng..

[84] Inkemia Green Chemicals Dibutyl Sebacate. https://shop.inkemiagreenchemicals.com/products/dibutyl-sebacate.

[85] European Chemicals Agency (2020). Dibutyl Sebacate Registration Dossier. https://echa.europa.eu/registration-dossier/-/registered-dossier/16127.

[86] Zhao G., Bi S., Li X., Wu J. (2010). Surface Tension of Diethyl Carbonate, 1,2-Dimethoxyethane and Diethyl Adipate. Fluid Phase Equilibria.

[87] European Chemicals Agency (2020). Diethyl Carbonate Registration Dossier. https://echa.europa.eu/registration-dossier/-/registered-dossier/14116.

[88] Thomsen M., Carlsen L., Hvidt S. (2001). Solubilities and Surface Activities of Phthalates Investigated by Surface Tension Measurements. Environ. Toxicol. Chem..

[89] European Chemicals Agency (2020). Diethyl Phthalate Registration Dossier. https://echa.europa.eu/registration-dossier/-/registered-dossier/14869.

[90] Ishigami T., Nii Y., Ohmukai Y., Rajabzadeh S., Matsuyama H. (2014). Solidification Behavior of Polymer Solution during Membrane Preparation by Thermally Induced Phase Separation. Membranes.

[91] Rajabzadeh S., Liang C., Ohmukai Y., Maruyama T., Matsuyama H. (2012). Effect of Additives on the Morphology and Properties of Poly(Vinylidene Fluoride) Blend Hollow Fiber Membrane Prepared by the Thermally Induced Phase Separation Method. J. Membr. Sci..

[92] Sherwood J., Bruyn M.D., Constantinou A., Moity L., McElroy C.R., Farmer T.J., Duncan T., Raverty W., Hunt A.J., Clark J.H. (2014). Dihydrolevoglucosenone (Cyrene) as a Bio-Based Alternative for Dipolar Aprotic Solvents. Chem. Commun..

[93] Salavagione H.J., Sherwood J., Bruyn M.D., Budarin V.L., Ellis G.J., Clark J.H., Shuttleworth P.S. (2017). Identification of High Performance Solvents for the Sustainable Processing of Graphene. Green Chem..

[94] European Chemicals Agency (2020). (1S,5R)-6,8-Dioxabicyclo[3.2.1]Octan-4-One Registration Dossier. https://echa.europa.eu/registration-dossier/-/registered-dossier/16252.

[95] Marino T., Galiano F., Molino A., Figoli A. (2019). New Frontiers in Sustainable Membrane Preparation: Cyrene™ as Green Bioderived Solvent. J. Membr. Sci..

[96] European Chemicals Agency (2020). N,N-Dimethylacetamide Registration Dossier. https://echa.europa.eu/registration-dossier/-/registered-dossier/15266.

[97] Gregorio R., Cestari M. (1994). Effect of Crystallization Temperature on the Crystalline Phase Content and Morphology of Poly(Vinylidene Fluoride). J. Polym. Sci. Part B Polym. Phys..

[98] Yeow M.L., Liu Y.T., Li K. (2004). Morphological Study of Poly(Vinylidene Fluoride) Asymmetric Membranes: Effects of the Solvent, Additive, and Dope Temperature. J. Appl. Polym. Sci..

[99] Otsuka T., Chujo Y. (2009). Synthesis of Transparent Poly(Vinylidene Fluoride) (PVdF)/Zirconium Oxide Hybrids without Crystallization of PVdF Chains. Polymer.

[100] Yu L.Y., Xu Z.L., Shen H.M., Yang H. (2009). Preparation and Characterization of PVDF–SiO2 Composite Hollow Fiber UF Membrane by Sol–Gel Method. J. Membr. Sci..

[101] Li Q., Xu Z.L., Yu L.Y. (2010). Effects of Mixed Solvents and PVDF Types on Performances of PVDF Microporous Membranes. J. Appl. Polym. Sci..

[102] Zhou X., He W., Li G., Zhang X., Zhu S., Huang J., Zhu S. Recycling of Electrode Materials from Spent Lithium-Ion Batteries. Proceedings of the 2010 4th International Conference on Bioinformatics and Biomedical Engineering.

[103] Wang Q., Wang Z., Wu Z. (2012). Effects of Solvent Compositions on Physicochemical Properties and Anti-Fouling Ability of PVDF Microfiltration Membranes for Wastewater Treatment. Desalination.

[104] Banerjee A., Kumar P.S., Shukla A.K. (2013). Influence of Binder Solvent on Carbon-Layer Structure in Electrical-Double-Layer Capacitors. J. Chem. Sci..

[105] Yi Z., Zhu L.P., Zhang H., Zhu B.K., Xu Y.Y. (2014). Ionic Liquids as Co-Solvents for Zwitterionic Copolymers and the Preparation of Poly(Vinylidene Fluoride) Blend Membranes with Dominated *β*-Phase Crystals. Polymer.

[106] Lee J., Park B., Kim J., Park S.B. (2015). Effect of PVP, Lithium Chloride, and Glycerol Additives on PVDF Dual-Layer Hollow Fiber Membranes Fabricated Using Simultaneous Spinning of TIPS and NIPS. Macromol. Res..

[107] Zhao Y., Zhou Y., Yang Y., Xu J., Chen Z.D., Jiang Y. (2018). The Impact of Solvents on Properties of Solution-Cast Poly(Vinylidene Fluoride) Films for Energy Storage. Mater. Lett..

[108] Mohammad A.A., Alkhaldi K.H.A.E., AlTuwaim M.S., Al-Jimaz A.S. (2013). Viscosity and Surface Tension of Binary Systems of N,N-Dimethylformamide with Alkan-1-Ols at Different Temperatures. J. Chem. Thermodyn..

[109] European Chemicals Agency (2020). N,N-Dimethylformamide Registration Dossier. https://echa.europa.eu/registration-dossier/-/registered-dossier/15093.

[110] Young T.H., Cheng L.P., Lin D.J., Fane L., Chuang W.Y. (1999). Mechanisms of PVDF Membrane Formation by Immersion-Precipitation in Soft (1-Octanol) and Harsh (Water) Nonsolvents. Polymer.

[111] Lin D.J., Beltsios K., Young T.H., Jeng Y.S., Cheng L.P. (2006). Strong Effect of Precursor Preparation on the Morphology of Semicrystalline Phase Inversion Poly(Vinylidene Fluoride) Membranes. J. Membr. Sci..

[112] Gregorio R. (2006). Determination of the *α*, *β*, and *γ* Crystalline Phases of Poly(Vinylidene Fluoride) Films Prepared at Different Conditions. J. Appl. Polym. Sci..

[113] Ma W., Zhang J., Chen S., Wang X. (2008). Crystalline Phase Formation of Poly(Vinylidene Fluoride) from Tetrahydrofuran/N,N-dimethylformamide Mixed Solutions. J. Macromol. Sci. Part B Phys..

[114] Gregorio R., Borges D.S. (2008). Effect of Crystallization Rate on the Formation of the Polymorphs of Solution Cast Poly(Vinylidene Fluoride). Polymer.

[115] Zhao X., Cheng J., Chen S., Zhang J., Wang X. (2010). Controlled Crystallization of Poly(Vinylidene Fluoride) Chains from Mixed Solvents Composed of Its Good Solvent and Nonsolvent. J. Polym. Sci. Part B Polym. Phys..

[116] Tao M.M., Liu F., Ma B.R., Xue L.X. (2013). Effect of Solvent Power on PVDF Membrane Polymorphism during Phase Inversion. Desalination.

[117] He L.P., Sun S.Y., Song X.F., Yu J.G. (2015). Recovery of Cathode Materials and Al from Spent Lithium-Ion Batteries by Ultrasonic Cleaning. Waste Manag..

[118] Kumar C., Viswanath P. (2017). Solvent Driven Polymorphism in Langmuir and Langmuir Schaefer Film of Poly(Vinylidene Fluoride). Eur. Polym. J..

[119] Natarajan S., Boricha A.B., Bajaj H.C. (2018). Recovery of Value-Added Products from Cathode and Anode Material of Spent Lithium-Ion Batteries. Waste Manag..

[120] Markarian S.A., Terzyan A.M. (2007). Surface Tension and Refractive Index of Dialkylsulfoxide + Water Mixtures at Several Temperatures. J. Chem. Eng. Data.

[121] European Chemicals Agency (2020). Dimethyl Sulfoxide Registration Dossier. https://echa.europa.eu/registration-dossier/-/registered-dossier/15007.

[122] Lin D.J., Chang C.L., Lee C.K., Cheng L.P. (2006). Preparation and Characterization of Microporous PVDF/PMMA Composite Membranes by Phase Inversion in Water/DMSO Solutions. Eur. Polym. J..

[123] Liu Z.H., Pan C.T., Lin L.W., Lai H.W. (2013). Piezoelectric Properties of PVDF/MWCNT Nanofiber Using near-Field Electrospinning. Sens. Actuators A Phys..

[124] Enayatzadeh M., Mohammadi T. (2018). Morphology and Performance of Poly(Vinylidene Fluoride) Flat Sheet Membranes: Thermodynamic and Kinetic Aspects. J. Appl. Polym. Sci..

[125] Rafati A.A., Ghasemian E., Iloukhani H. (2009). Surface Tension and Surface Properties of Binary Mixtures of 1,4-Dioxane or N,N-Dimethyl Formamide with n-Alkyl Acetates. J. Chem. Eng. Data.

[126] European Chemicals Agency (2020). 1,4-Dioxane Registration Dossier. https://echa.europa.eu/registration-dossier/-/registered-dossier/15842.

[127] Lee M.K., Lee J. (2015). Mimicking Permafrost Formation for the Preparation of Porous Polymer Membranes. Polymer.

[128] Kirk-Othmer (1981). Kirk-Othmer Encyclopedia of Chemical Technology.

[129] Yaws C.L., Richmond P.C., Yaws C.L. (2009). Chapter 21—Surface Tension—Organic Compounds. Thermophysical Properties of Chemicals and Hydrocarbons.

[130] ChemBK (2015). HEPTAN-3-ONE. https://www.chembk.com/en/chem/HEPTAN-3-ONE.

[131] Taniewska-Osińska S., Jóźwiak M. (1989). Densimetric and Viscosimetric Investigations of NaI in Hexamethylphosphoramide–Water Mixtures at 293.15, 298.15 and 303.15 K. J. Chem. Soc. Faraday Trans. Phys. Chem. Condens. Phases.

[132] Sigma-Aldrich (2020). Hexamethylphosphoramide. https://www.sigmaaldrich.com/catalog/product/aldrich/52730.

[133] Ha D.M. (2013). Measurement and Prediction of Fire and Explosion Properties of 3-Hexanone. J. Korean Inst. Gas.

[134] Sigma-Aldrich (2020). 3-Hexanone. https://www.sigmaaldrich.com/catalog/product/aldrich/103020.

[135] U.S. EPA (2020). 3-Hexanone. https://comptox.epa.gov/dashboard/dsstoxdb/results?search=DTXSID2021608.

[136] Ouyang G., Yang Y., Lu S., Huang Z., Kang B. (2004). Excess Molar Volumes and Surface Tensions of Xylene with Acetone or 2-Butanone at 298.15 K. J. Chem. Eng. Data.

[137] European Chemicals Agency (2020). Butanone Registration Dossier. https://echa.europa.eu/registration-dossier/-/registered-dossier/15065.

[138] Kahl H., Wadewitz T., Winkelmann J. (2003). Surface Tension of Pure Liquids and Binary Liquid Mixtures. J. Chem. Eng. Data.

[139] European Chemicals Agency (2020). 1-Methyl-2-Pyrrolidone Registration Dossier. https://echa.europa.eu/registration-dossier/-/registered-dossier/15493.

[140] Contestabile M., Panero S., Scrosati B. (2001). A Laboratory-Scale Lithium-Ion Battery Recycling Process. J. Power Sources.

[141] Sukitpaneenit P., Chung T.S. (2009). Molecular Elucidation of Morphology and Mechanical Properties of PVDF Hollow Fiber Membranes from Aspects of Phase Inversion, Crystallization and Rheology. J. Membr. Sci..

[142] Sun A.C., Kosar W., Zhang Y., Feng X. (2013). A Study of Thermodynamics and Kinetics Pertinent to Formation of PVDF Membranes by Phase Inversion. Desalination.

[143] Zhang X., Xie Y., Lin X., Li H., Cao H. (2013). An Overview on the Processes and Technologies for Recycling Cathodic Active Materials from Spent Lithium-Ion Batteries. J. Mater. Cycles Waste Manag..

[144] Xu H.P., Lang W.Z., Zhang X., Guo Y.J. (2015). Preparation and Characterizations of Charged PVDF Membranes via Composite Thermally Induced Phase Separation (C-TIPS) Method. J. Ind. Eng. Chem..

[145] European Chemicals Agency (2020). Octan-3-One Registration Dossier. https://echa.europa.eu/registration-dossier/-/registered-dossier/22922.

[146] Abbott S. (2015). Hansen Solubility Parameters in Practice—Complete with Software, Data, and Examples.

[147] Randová A., Bartovská L., Morávek P., Matějka P., Novotná M., Matějková S., Drioli E., Figoli A., Lanč M., Friess K. (2016). A Fundamental Study of the Physicochemical Properties of Rhodiasolv^®^Polarclean: A Promising Alternative to Common and Hazardous Solvents. J. Mol. Liq..

[148] Hassankiadeh N.T., Cui Z., Kim J.H., Shin D.W., Lee S.Y., Sanguineti A., Arcella V., Lee Y.M., Drioli E. (2015). Microporous Poly(Vinylidene Fluoride) Hollow Fiber Membranes Fabricated with PolarClean as Water-Soluble Green Diluent and Additives. J. Membr. Sci..

[149] Jung J.T., Kim J.F., Wang H.H., Di Nicolo E., Drioli E., Lee Y.M. (2016). Understanding the Non-Solvent Induced Phase Separation (NIPS) Effect during the Fabrication of Microporous PVDF Membranes via Thermally Induced Phase Separation (TIPS). J. Membr. Sci..

[150] Lide D. (2005). (Ed.) CRC Handbook of Chemistry and Physics.

[151] European Chemicals Agency (2020). Pentan-3-One Registration Dossier. https://echa.europa.eu/registration-dossier/-/registered-dossier/2213.

[152] European Chemicals Agency (2020). Propylene Carbonate Registration Dossier. https://echa.europa.eu/registration-dossier/-/registered-dossier/16088.

[153] Naejus R., Lemordant D., Coudert R., Willmann P. (1997). Excess Thermodynamic Properties of Binary Mixtures Containing Linear or Cyclic Carbonates as Solvents at the Temperatures 298.15 K and 315.15 K. J. Chem. Thermodyn..

[154] Tahery R. (2017). Surface Tension Measurements for Binary Mixtures of Chlorobenzene or Chlorocyclohexane + Tetrahydrofuran at 298.15 K. J. Solut. Chem..

[155] European Chemicals Agency (2020). Tetrahydrofuran Registration Dossier. https://echa.europa.eu/registration-dossier/-/registered-dossier/15474.

[156] Caruso J.A., Barker B.J. (1971). Solvation and Chemical Equilibrium Studies of Alkali Metal Salts in 1,1,3,3-Tetramethylurea. J. Am. Chem. Soc..

[157] Lindfors K.R., Opperman S.H., Glover M.E., Seese J.D. (1971). Intermolecular Hydrogen Bonding. I. Effects on the Physical Properties of Tetramethylurea-Water Mixtures. J. Phys. Chem..

[158] U.S. EPA (2020). Tetramethylurea. https://comptox.epa.gov/dashboard/dsstoxdb/results?search=DTXSID1060893.

[159] Benerito R.R., Singleton W.S., Feuge R.O. (1954). Surface and Interfacial Tensions of Synthetic Glycerides of Known Composition and Configuration. J. Phys. Chem..

[160] European Chemicals Agency (2020). Triacetin Registration Dossier. https://echa.europa.eu/registration-dossier/-/registered-dossier/15139.

[161] Rajabzadeh S., Maruyama T., Sotani T., Matsuyama H. (2008). Preparation of PVDF Hollow Fiber Membrane from a Ternary Polymer/Solvent/Nonsolvent System via Thermally Induced Phase Separation (TIPS) Method. Sep. Purif. Technol..

[162] Rajabzadeh S., Teramoto M., Al-Marzouqi M.H., Kamio E., Ohmukai Y., Maruyama T., Matsuyama H. (2010). Experimental and Theoretical Study on Propylene Absorption by Using PVDF Hollow Fiber Membrane Contactors with Various Membrane Structures. J. Membr. Sci..

[163] Rajabzadeh S., Maruyama T., Ohmukai Y., Sotani T., Matsuyama H. (2009). Preparation of PVDF/PMMA Blend Hollow Fiber Membrane via Thermally Induced Phase Separation (TIPS) Method. Sep. Purif. Technol..

[164] Ghasem N., Al-Marzouqi M., Duaidar A. (2011). Effect of Quenching Temperature on the Performance of Poly(Vinylidene Fluoride) Microporous Hollow Fiber Membranes Fabricated via Thermally Induced Phase Separation Technique on the Removal of CO2 from CO2-Gas Mixture. Int. J. Greenh. Gas Control.

[165] European Chemicals Agency (2020). Triethyl Citrate Registration Dossier. https://echa.europa.eu/registration-dossier/-/registered-dossier/14584.

[166] Kannan S., Kishore K. (1999). Absolute Viscosity and Density of Trisubstituted Phosphoric Esters. J. Chem. Eng. Data.

[167] European Chemicals Agency (2019). Triethyl Phosphate Substance Information. https://echa.europa.eu/substance-information/-/substanceinfo/100.001.013.

[168] Zhang Z., Guo C., Li X., Liu G., Lv J. (2013). Effects of PVDF Crystallization on Polymer Gelation Behavior and Membrane Structure from PVDF/TEP System via Modified TIPS Process. Polym. Plast. Technol. Eng..

[169] Chang J., Zuo J., Zhang L., O’Brien G.S., Chung T.S. (2017). Using Green Solvent, Triethyl Phosphate (TEP), to Fabricate Highly Porous PVDF Hollow Fiber Membranes for Membrane Distillation. J. Membr. Sci..

[170] Marino T., Russo F., Figoli A. (2018). The Formation of Polyvinylidene Fluoride Membranes with Tailored Properties via Vapour/Non-Solvent Induced Phase Separation. Membranes.

[171] European Chemicals Agency (2020). Trimethyl Phosphate Registration Dossier. https://echa.europa.eu/registration-dossier/-/registered-dossier/22505.

[172] Byrne F.P., Nussbaumer C.M., Savin E.J., Milescu R.A., McElroy C.R., Clark J.H., Van Vugt-Lussenburg B.M.A., Van der Burg B., Meima M.Y., Buist H.E. (2020). A Family of Water-Immiscible, Dipolar Aprotic, Diamide Solvents from Succinic Acid. ChemSusChem.

[173] European Chemicals Agency (2020). Acetophenone Registration Dossier. https://echa.europa.eu/registration-dossier/-/registered-dossier/14683/4/23.

[174] Mal S., Nandi A.K. (1998). A Thermodynamic Study on the Thermoreversible Poly(Vinylidene Fluoride) Gels in Acetophenone, Ethyl Benzoate, and Glyceryl Tributyrate. Langmuir.

[175] Mal S., Nandi A.K. (1999). Influence of Chain Structure and Molecular Weight of Poly(Vinylidene Fluoride) on the Morphology of Its Thermoreversible Gels in Acetophenone, Ethyl Benzoate, and Glyceryl Tributyrate. Macromol. Chem. Phys..

[176] European Chemicals Agency (2019). Tributyl O-Acetylcitrate Substance Information. https://echa.europa.eu/substance-information/-/substanceinfo/100.000.971.

[177] Cui Z., Hassankiadeh N.T., Lee S.Y., Lee J.M., Woo K.T., Sanguineti A., Arcella V., Lee Y.M., Drioli E. (2013). Poly(Vinylidene Fluoride) Membrane Preparation with an Environmental Diluent via Thermally Induced Phase Separation. J. Membr. Sci..

[178] Hassankiadeh N.T., Cui Z., Kim J.H., Shin D.W., Sanguineti A., Arcella V., Lee Y.M., Drioli E. (2014). PVDF Hollow Fiber Membranes Prepared from Green Diluent via Thermally Induced Phase Separation: Effect of PVDF Molecular Weight. J. Membr. Sci..

[179] European Chemicals Agency (2020). Benzophenone Registration Dossier. https://echa.europa.eu/registration-dossier/-/registered-dossier/13823/4/3.

[180] Gu M., Zhang J., Xia Y., Wang X. (2008). Poly(Vinylidene Fluoride) Crystallization Behavior and Membrane Structure Formation Via Thermally Induced Phase Separation with Benzophenone Diluent. J. Macromol. Sci. Part B.

[181] Yang J., Li D.W., Lin Y.K., Wang X.L., Tian F., Wang Z. (2008). Formation of a Bicontinuous Structure Membrane of Polyvinylidene Fluoride in Diphenyl Ketone Diluent via Thermally Induced Phase Separation. J. Appl. Polym. Sci..

[182] Lin Y., Tang Y., Ma H., Yang J., Tian Y., Ma W., Wang X. (2009). Formation of a Bicontinuous Structure Membrane of Polyvinylidene Fluoride in Diphenyl Carbonate Diluent via Thermally Induced Phase Separation. J. Appl. Polym. Sci..

[183] Tang Y., Lin Y., Ma W., Tian Y., Yang J., Wang X. (2010). Preparation of Microporous PVDF Membrane via Tips Method Using Binary Diluent of DPK and PG. J. Appl. Polym. Sci..

[184] Kim K.S., Demberelnyamba D., Shin B.K., Yeon S.H., Choi S., Cha J.H., Lee H., Lee C.S., Shim J.J. (2006). Surface Tension and Viscosity of 1-Butyl-3-Methylimidazolium Iodide and 1-Butyl-3-Methylimidazolium Tetrafluoroborate, and Solubility of Lithium Bromide+1-Butyl-3-Methylimidazolium Bromide in Water. Korean J. Chem. Eng..

[185] Tian S., Hou Y., Wu W., Ren S., Pang K. (2012). Physical Properties of 1-Butyl-3-Methylimidazolium Tetrafluoroborate/N-Methyl-2-Pyrrolidone Mixtures and the Solubility of CO2 in the System at Elevated Pressures. J. Chem. Eng. Data.

[186] Sigma-Aldrich (2020). 1-Butyl-3-Methylimidazolium Tetrafluoroborate. https://www.sigmaaldrich.com/catalog/product/sial/39931.

[187] Zeng X., Li J. (2014). Innovative Application of Ionic Liquid to Separate Al and Cathode Materials from Spent High-Power Lithium-Ion Batteries. J. Hazard. Mater..

[188] Sigma-Aldrich (2020). Epsilon-Caprolactam. https://www.sigmaaldrich.com/catalog/product/aldrich/c2204?lang=en&region=GB.

[189] European Chemicals Agency (2020). ϵ-Caprolactam Registration Dossier. https://echa.europa.eu/registration-dossier/-/registered-dossier/15939/4/3.

[190] Liu Z.H., Maréchal P., Jérôme R. (1996). Intermolecular Interactions in Poly(Vinylidene Fluoride) and *ϵ*-Caprolactam Mixtures. Polymer.

[191] Bürer T., Günthard H. (1957). Infrarot-Spektren von Cyclanen Und Cyclanonen. III. Flüssigkeits- Und Festkörperspektren Der Cyclanone. Helv. Chim. Acta.

[192] Sigma-Aldrich (2020). Cycloheptanone. https://www.sigmaaldrich.com/catalog/product/aldrich/c99000?lang=en&region=GB.

[193] European Chemicals Agency (2020). Dimethyl Adipate Registration Dossier. https://echa.europa.eu/registration-dossier/-/registered-dossier/14132/4/11.

[194] Dikshit A.K., Nandi A.K. (1998). Thermoreversible Gelation of Poly(Vinylidene Fluoride) in Diethyl Adipate: A Concerted Mechanism. Macromolecules.

[195] Dasgupta D., Nandi A.K. (2005). Multiporous Polymeric Materials from Thermoreversible Poly(Vinylidene Fluoride) Gels. Macromolecules.

[196] Sigma-Aldrich (2020). Diethyl Azelate. https://www.sigmaaldrich.com/catalog/product/aldrich/124583.

[197] U.S. EPA (2020). Diethyl Azelate. https://comptox.epa.gov/dashboard/dsstoxdb/results?search=DTXSID4060783.

[198] Dikshit A.K., Nandi A.K. (2000). Thermoreversible Gelation of Poly(Vinylidene Fluoride) in Diesters: Influence of Intermittent Length on Morphology and Thermodynamics of Gelation. Macromolecules.

[199] Sigma-Aldrich (2020). Diethyl Glutarate. https://www.sigmaaldrich.com/catalog/product/aldrich/d96006.

[200] U.S. EPA (2020). Diethyl Glutarate. https://comptox.epa.gov/dashboard/dsstoxdb/results?search=DTXSID5061162.

[201] Venkateswararao V., Satyanarayana G., Beebi S., Rambabu C. (2018). Thermo-Physical Studies on Molecular Interactions in Liquid Binaries of Diethyl Malonate and Isomeric Xylenes at Various Temperatures. Der Pharma Chem..

[202] European Chemicals Agency (2020). Diethyl Malonate Registration Dossier. https://echa.europa.eu/registration-dossier/-/registered-dossier/5774.

[203] European Chemicals Agency (2020). Diethyl Oxalate Registration Dossier. https://echa.europa.eu/registration-dossier/-/registered-dossier/14261/4/23.

[204] Sigma-Aldrich (2020). Diethyl Pimelate. https://www.sigmaaldrich.com/catalog/product/aldrich/d99706.

[205] U.S. EPA (2020). Diethyl Pimelate. https://comptox.epa.gov/dashboard/dsstoxdb/results?search=DTXSID30174485.

[206] Wang S., Bi S., Wu J. (2017). Surface Tension of Four Oxygenated Fuels: Experiment and Correlation. Fluid Phase Equilibria.

[207] European Chemicals Agency (2020). Diethyl Succinate Registration Dossier. https://echa.europa.eu/registration-dossier/-/registered-dossier/27981/4/11.

[208] European Chemicals Agency (2020). Dimethyl Phthalate Registration Dossier. https://echa.europa.eu/registration-dossier/-/registered-dossier/14997.

[209] Gu M., Zhang J., Wang X., Ma W. (2006). Crystallization Behavior of PVDF in PVDF-DMP System via Thermally Induced Phase Separation. J. Appl. Polym. Sci..

[210] European Chemicals Agency (2020). Dimethyl Sulphone Registration Dossier. https://echa.europa.eu/registration-dossier/-/registered-dossier/17580.

[211] Liang H.Q., Wu Q.Y., Wan L.S., Huang X.J., Xu Z.K. (2013). Polar Polymer Membranes via Thermally Induced Phase Separation Using a Universal Crystallizable Diluent. J. Membr. Sci..

[212] Sigma-Aldrich (2020). Diphenyl Carbonate. https://www.sigmaaldrich.com/catalog/product/aldrich/d206539.

[213] U.S. EPA (2020). Diphenylcarbonate. https://comptox.epa.gov/dashboard/dsstoxdb/results?search=DTXSID3020540.

[214] European Chemicals Agency (2020). Ethyl Benzoate Registration Dossier. https://echa.europa.eu/registration-dossier/-/registered-dossier/16489/4/23.

[215] Rodriguez M., Galan M., Munoz M.J., Martin R. (1994). Viscosity of Triglycerides + Alcohols from 278 to 313 K. J. Chem. Eng. Data.

[216] O’Neil M. (2001). The Merck Index.

[217] Mal S., Nandi A.K. (1998). Gelation Mechanism of Thermoreversible Poly(Vinylidene Fluoride) Gels in Glyceryl Tributyrate. Polymer.

[218] European Chemicals Agency (2020). Methyl Salicylate Registration Dossier. https://echa.europa.eu/registration-dossier/-/registered-dossier/2227/4/23.

[219] Ma W., Chen S., Zhang J., Wang X. (2010). Kinetics of Thermally Induced Phase Separation in the PVDF Blend/Methyl Salicylate System and Its Effect on Membrane Structures. J. Macromol. Sci. Part B.

[220] European Chemicals Agency (2019). Sulfolane Registration Dossier. https://echa.europa.eu/registration-dossier/-/registered-dossier/13657.

[221] Cui Z.Y., Xu Y.Y., Zhu L.P., Wei X.Z., Zhang C.F., Zhu B.K. (2008). Preparation of PVDF/PMMA Blend Microporous Membranes for Lithium Ion Batteries via Thermally Induced Phase Separation Process. Mater. Lett..

[222] Sigman M.E., Lindley S.M., Leffler J.E. (1985). Supercritical Carbon Dioxide: Behavior of .Pi.* and .Beta. Solvatochromic Indicators in Media of Different Densities. J. Am. Chem. Soc..

[223] Ouyang L.B. (2011). New Correlations for Predicting the Density and Viscosity of Supercritical Carbon Dioxide Under Conditions Expected in Carbon Capture and Sequestration Operations. Open Pet. Eng. J..

[224] Lora M., Lim J.S., McHugh M.A. (1999). Comparison of the Solubility of PVF and PVDF in Supercritical CH2F2 and CO2 and in CO2 with Acetone, Dimethyl Ether, and Ethanol. J. Phys. Chem. B.

[225] Dinoia T.P., Conway S.E., Lim J.S., McHugh M.A. (2000). Solubility of Vinylidene Fluoride Polymers in Supercritical CO2 and Halogenated Solvents. J. Polym. Sci. Part B Polym. Phys..

[226] Cui Z., Hassankiadeh N.T., Lee S.Y., Woo K.T., Lee J.M., Sanguineti A., Arcella V., Lee Y.M., Drioli E. (2015). Tailoring Novel Fibrillar Morphologies in Poly(Vinylidene Fluoride) Membranes Using a Low Toxic Triethylene Glycol Diacetate (TEGDA) Diluent. J. Membr. Sci..

[227] Miller-Chou B.A., Koenig J.L. (2003). A Review of Polymer Dissolution. Prog. Polym. Sci..

[228] Rasool M.A., Vankelecom I.F.J. (2019). Use of *γ*-Valerolactone and Glycerol Derivatives as Bio-Based Renewable Solvents for Membrane Preparation. Green Chem..

[229] Rasool M.A., Pescarmona P.P., Vankelecom I.F.J. (2019). Applicability of Organic Carbonates as Green Solvents for Membrane Preparation. ACS Sustain. Chem. Eng..

[230] Kim J.F., Kim J.H., Lee Y.M., Drioli E. (2016). Thermally Induced Phase Separation and Electrospinning Methods for Emerging Membrane Applications: A Review. AIChE J..

[231] Tan X.M., Rodrigue D. (2019). A review on porous polymeric membrane preparation. Part I: Production techniques with polysulfone and poly (vinylidene fluoride). Polymers.

[232] Liu F., Hashim N.A., Liu Y., Abed M.R.M., Li K. (2011). Progress in the Production and Modification of PVDF Membranes. J. Membr. Sci..

[233] Guillen G.R., Pan Y., Li M., Hoek E.M.V. (2011). Preparation and Characterization of Membranes Formed by Nonsolvent Induced Phase Separation: A Review. Ind. Eng. Chem. Res..

[234] Wang D.M., Lai J.Y. (2013). Recent Advances in Preparation and Morphology Control of Polymeric Membranes Formed by Nonsolvent Induced Phase Separation. Curr. Opin. Chem. Eng..

[235] Capello C., Fischer U., Hungerbühler K. (2007). What is a green solvent? A comprehensive framework for the environmental assessment of solvents. Green Chem..

[236] Byrne F.P., Jin S., Paggiola G., Petchey T., Clark J.H., Farmer T.J., Hunt A.J., McElroy C.R., Sherwood J. (2016). Tools and techniques for solvent selection: Green solvent selection guides. Sustain. Chem. Process..

[237] European Chemicals Agency (2019). Dimethyl Sulfoxide Substance Information. https://echa.europa.eu/substance-information/-/substanceinfo/100.000.604.

[238] Figoli A., Marino T., Simone S., Nicolò E.D., Li X.M., He T., Tornaghi S., Drioli E. (2014). Towards Non-Toxic Solvents for Membrane Preparation: A Review. Green Chem..

[239] Glindemann D., Novak J., Witherspoon J. (2006). Dimethyl Sulfoxide (DMSO) Waste Residues and Municipal Waste Water Odor by Dimethyl Sulfide (DMS): The North-East WPCP Plant of Philadelphia. Environ. Sci. Technol..

[240] Strathmann H., Kock K. (1977). The formation mechanism of phase inversion membranes. Desalination.

[241] Fiume M.Z., Cosmetic Ingredients Review Expert Panel (2003). Final report on the safety assessment of triacetin. Int. J. Toxicol..

[242] Ghasem N., Al-Marzouqi M., Duidar A. (2012). Effect of PVDF concentration on the morphology and performance of hollow fiber membrane employed as gas–liquid membrane contactor for CO2 absorption. Sep. Purif. Technol..

[243] Camp J.E. (2018). Bio-Available Solvent Cyrene: Synthesis, Derivatization, and Applications. ChemSusChem.

[244] Circa (2018). Press Release: Circa Receives Green Light to Sell Non-Toxic, Bio-Based and Biodegradable Solvent in EU. https://www.sustainabilityconsult.com/news/361-press-release-circa-receives-green-light-to-sell-non-toxic-bio-based-and-biodegradable-solvent-in-eu.

[245] European Chemicals Agency (2019). (1S,5R)-6,8-Dioxabicyclo[3.2.1]Octan-4-One Substance Information. https://echa.europa.eu/substance-information/-/substanceinfo/100.234.612.

[246] European Chemicals Agency (2019). 2,2’-[Ethane-1,2-Diylbis(Oxy)]Bisethyl Diacetate Substance Information. https://echa.europa.eu/substance-information/-/substanceinfo/100.003.497.

[247] Solvay (2021). Rhodiasolv. https://www.solvay.com/en/brands/rhodiasolv-polarclean.

[248] Cadman P., Gossedge G.M. (1979). The chemical interaction of metals with polytetrafluoroethylene. J. Mater. Sci..

[249] Madorsky S.L., Straus S. (1959). Thermal Degradation of Polymers at High Temperatures. J. Res. Natl. Bur. Stand. Sect. Phys. Chem..

[250] Nguyen T. (1985). Degradation of Poly[vinyl Fluoride] and Poly[vinylidene Fluoride]. J. Macromol. Sci. Part C.

[251] Slater P. (2002). Poly(vinylidene fluoride) as a reagent for the synthesis of K2NiF4-related inorganic oxide fluorides. J. Fluor. Chem..

[252] Wang J., Shin Y., Arenholz E., Lefler B.M., Rondinelli J.M., May S.J. (2018). Effect of fluoropolymer composition on topochemical synthesis of *SrMn*O_3-δ_F_γ_ oxyfluoride films. Phys. Rev. Mater..

[253] Wentink T., Willwerth L.J., Phaneuf J.P. (1961). Properties of polyvinylidene fluoride. Part II. Infrared transmission of normal and thermally decomposed polymer. J. Polym. Sci..

[254] Badali Y., Kcoyigit S., Aytimur A., Altindal S., Uslu I. (2019). Synthesis of boron and rare earth stabilized graphene doped polyvinylidene fluoride (PVDF) nanocomposite piezoelectric materials. Polym. Compos..

[255] Ouyang Z.W., Chen E.C., Wu T.M. (2015). Thermal stability and magnetic properties of polyvinylidene fluoride/magnetite nanocomposites. Materials.

[256] Li X., Huang C., Yang H., Li Y., Cheng Y. (2016). Thermal reaction properties of aluminum/copper (II) oxide/poly(vinylidene fluoride) nanocomposite. J. Therm. Anal. Calorim..

[257] Liu F., Moghareh Abed M.R., Li K. (2011). Preparation and characterization of poly(vinylidene fluoride) (PVDF) based ultrafiltration membranes using nano *γ*-Al_2_O_3_. J. Membr. Sci..

[258] Li H., Kim H. (2008). Thermal degradation and kinetic analysis of PVDF/modified MMT nanocomposite membranes. Desalination.

[259] Shen Y., Lua A.C. (2012). Preparation and characterization of mixed matrix membranes based on PVDF and three inorganic fillers (fumed nonporous silica, zeolite 4A and mesoporous MCM-41) for gas separation. Chem. Eng. J..

[260] Teow Y.H., Latif A.A., Lim J.K., Ngang H.P., Susan L.Y., Ooi B.S. (2015). Hydroxyl functionalized PVDF-TiO_2_ ultrafiltration membrane and its antifouling properties. J. Appl. Polym. Sci..

[261] Bei P., Liu H., Yao H., Hu A., Sun Y., Guo L. (2019). Preparation and characterization of PVDF/CaCO_3_ composite membranes etched by hydrochloric acid. Environ. Sci. Pollut. Res..

[262] Xiao L., Davenport D.M., Ormsbee L., Bhattacharyya D. (2015). Polymerization and Functionalization of Membrane Pores for Water Related Applications. Ind. Eng. Chem. Res..

[263] Wootthikanokkhan J., Changsuwan P. (2008). Dehydrofluorination of PVDF and Proton Conductivity of the Modified PVDF/Sulfonated SEBS Blend Membranes. J. Met. Mater. Miner..

[264] Ross G.J., Watts J.F., Hill M.P., Morrissey P. (2000). Surface modification of poly(vinylidene fluoride) by alkaline treatment: 1. The degradation mechanism. Polymer.

[265] Kise H., Ogata H., Nakata M. (1989). Chemical dehydrofluorination and electrical conductivity of poly(vinylidene fluoride) films. Die Angew. Makromol. Chem..

[266] Kise H., Ogata H. (1983). Phase Transfer Catalysis in Dehydrofluorination of Poly(Vinylidene Fluoride) By Aqueous Sodium Hydroxide Solutions. J. Polym. Sci. Part A Polym. Chem..

[267] Taguet A., Ameduri B., Boutevin B. (2005). Crosslinking of vinylidene fluoride-containing fluoropolymers. Adv. Polym. Sci..

[268] Goethem C.V., Mertens M., Vankelecom I.F.J. (2019). Crosslinked PVDF membranes for aqueous and extreme pH nano filtration. J. Membr. Sci..

[269] Rabuni M.F., Nik Sulaiman N.M., Aroua M.K., Hashim N.A. (2013). Effects of alkaline environments at mild conditions on the stability of PVDF membrane: An experimental study. Ind. Eng. Chem. Res..

[270] Li D., Liao M. (2017). Dehydrofluorination mechanism, structure and thermal stability of pure fluoroelastomer (poly(VDF-ter-HFP-ter-TFE) terpolymer) in alkaline environment. J. Fluor. Chem..

[271] Zhao X., Niketic S., Yim C.H., Zhou J., Wang J., Abu-Lebdeh Y. (2018). Revealing the Role of Poly(vinylidene fluoride) Binder in Si/Graphite Composite Anode for Li-Ion Batteries. ACS Omega.

[272] Dias A.J., Mccarthy T.J. (1985). Dehydrofluorination of Poly (vinylidene Fluoride) in Dimethylformamide Solution: Synthesis of an Operationally Soluble Semiconducting Polymer. J. Polym. Sci. Polym. Chem. Ed..

[273] Brewis D.M., Mathieson I., Sutherland I., Cayless R.A., Dahm R.H. (1996). Pretreatment of poly(vinyl fluoride) and poly(vinylidene fluoride) with potassium hydroxide. Int. J. Adhes. Adhes..

[274] Samsure N.A., Hashim N.A., Nik Sulaiman N.M., Chee C.Y. (2016). Alkaline etching treatment of PVDF membrane for water filtration. RSC Adv..

[275] Pagliaro L., Lowy D.A. (2019). Interaction of Polyvinylidene Fluoride (PVDF)-Based Binders With Strongly Alkaline Solutions. Green Chem..

[276] Wu C., Tang W., Zhang J., Liu S., Wang Z., Wang X., Lu X. (2017). Preparation of super-hydrophobic PVDF membrane for MD purpose via hydroxyl induced crystallization-phase inversion. J. Membr. Sci..

[277] Cai X., Lei T., Sun D., Lin L. (2017). A critical analysis of the *α*, *β* and *γ* phases in poly(vinylidene fluoride) using FTIR. RSC Adv..

[278] Abed A., Bouazizi N., Giraud S., El A., Campagne C. (2020). Polyester-supported Chitosan-Poly (vinylidene fluoride)-Inorganic- Oxide-Nanoparticles Composites with Improved Flame Retardancy. Chin. J. Polym. Sci..

[279] Han B., Piernas-Muñoz M.J., Dogan F., Kubal J., Trask S.E., Bloom I.D., Vaughey J.T., Key B. (2019). Probing the reaction between PVDF and LiPAA vs Li7Si3: Investigation of binder stability for si anodes. J. Electrochem. Soc..

[280] Papp J.K., Forster J.D., Burke C.M., Kim H.W., Luntz A.C., Shelby R.M., Urban J.J., McCloskey B.D. (2017). Poly(vinylidene fluoride) (PVDF) Binder Degradation in Li–O2 Batteries: A Consideration for the Characterization of Lithium Superoxide. J. Phys. Chem. Lett..

[281] Lee M.J., Ong C.S., Lau W.J., Ng B.C., Ismail A.F., Lai S.O. (2016). Degradation of PVDF-based composite membrane and its impacts on membrane intrinsic and separation properties. J. Polym. Eng..

[282] Ke X., Zhang Y., Gohs U., Drache M., Beuermann S. (2019). Polymer Electrolyte Membranes Prepared by Graft Copolymerization of 2-Acrylamido-2-Methylpropane Sulfonic Acid and Acrylic Acid on PVDF and ETFE Activated by Electron Beam Treatment. Polymers.

[283] Danks T.N., Slade R.C., Varcoe J.R. (2003). Alkaline anion-exchange radiation-grafted membranes for possible electrochemical application in fuel cells. J. Mater. Chem..

[284] Adem E., Rickards J., Burillo G., Avalos-Borja M. (1999). Changes in poly-vinylidene fluoride produced by electron irradiation. Radiat. Phys. Chem..

[285] Nasef M.M., Saidi H., Dahlan K.Z.M. (2002). Investigation of electron irradiation induced-changes in poly(vinylidene fluoride) films. Polym. Degrad. Stab..

[286] Jaleh B., Gavar N., Fakhri P., Muensit N., Taheri S.M. (2015). Characteristics of PVDF membranes irradiated by electron beam. Membranes.

[287] Lim Y.M., Kang P.H., Lee S.M., Kim S.S., Jeun J.P., Jung C.H., Choi J.H., Lee Y.M., Nho Y.C. (2006). Effect of electron beam irradiation on poly(vinylidene fluoride) films at the melting temperature. J. Ind. Eng. Chem..

[288] Medeiros A.S., Gual M.R., Pereira C., Faria L.O. (2015). Thermal analysis for study of the gamma radiation effects in poly(vinylidene fluoride). Radiat. Phys. Chem..

